# Predictors of CD4 cell recovery following initiation of antiretroviral therapy among HIV‐1 positive patients with well‐estimated dates of seroconversion

**DOI:** 10.1111/hiv.12567

**Published:** 2017-12-01

**Authors:** OT Stirrup, AJ Copas, AN Phillips, MJ Gill, RB Geskus, G Touloumi, J Young, HC Bucher, AG Babiker, CASCADE Collaboration in EuroCoord, Tony Kelleher, David Cooper, Pat Grey, Robert Finlayson, Mark Bloch, Tony Kelleher, Tim Ramacciotti, Linda Gelgor, Don Smith, Robert Zangerle, John Gill, Irja Lutsar, Geneviève Chêne, Francois Dabis, Rodolphe Thiebaut, Dominique Costagliola, Marguerite Guiguet, Philippe Vanhems, Marie‐Laure Chaix, Jade Ghosn, Laurence Meyer, Faroudy Boufassa, Osamah Hamouda, Karolin Meixenberger, Norbert Bannert, Barbara Bartmeyer, Anastasia Antoniadou, Georgios Chrysos, Georgios L. Daikos, Nikos Pantazis, Olga Katsarou, Giovanni Rezza, Maria Dorrucci, Antonella Monforte, Andrea Luca, Maria Prins, Ronald Geskus, Jannie Helm, Hanneke Schuitemaker, Mette Sannes, Oddbjorn Brubakk, Anne‐Marte Kran, Magdalena Rosinska, Roberto Muga, Jordi Tor, Patricia Olalla, Joan Cayla, Julia Amo, Santiago Moreno, Susana Monge, Julia Amo, Jorge Romero, Santiago Pérez‐Hoyos, Anders Sönnerborg, C Bucher, Huldrych Günthard, Alexandra Scherrer, Ruslan Malyuta, Gary Murphy, Kholoud Porter, Anne Johnson, Abdel Babiker, Deenan Pillay, Charles Morrison, Robert Salata, Roy Mugerwa, Tsungai Chipato, Matt A. Price, Jill Gilmour, Anatoli Kamali, Etienne Karita

**Affiliations:** ^1^ MRC Clinical Trials Unit University College London London UK; ^2^ Research Department of Infection & Population Health University College London London UK; ^3^ Department of Medicine University of Calgary Calgary AB Canada; ^4^ Department of Clinical Epidemiology, Biostatistics and Bioinformatics Academic Medical Center (AMC) Amsterdam The Netherlands; ^5^ Department of Infectious Diseases Public Health Service of Amsterdam Amsterdam The Netherlands; ^6^ Oxford University Clinical Research Unit Wellcome Trust Major Overseas Programme Ho Chi Minh City Vietnam; ^7^ Nuffield Department of Clinical Medicine Centre for Tropical Medicine and Global Health University of Oxford Oxford UK; ^8^ Department of Hygiene, Epidemiology and Medical Statistics, Medical School National and Kapodistrian University of Athens Athens Greece; ^9^ Basel Institute for Clinical Epidemiology and Biostatistics University Hospital Basel and University of Basel Basel Switzerland

**Keywords:** antiretroviral therapy, ART, CD4, HAART, HIV, longitudinal data, mixed effects model

## Abstract

**Objectives:**

To investigate factors that predict speed of recovery and long‐term CD4 cell count in HIV‐1 seroconverters initiating combination antiretroviral therapy (cART), and to quantify the influence of very early treatment initiation. We make use of all pre‐treatment CD4 counts, because analyses using only a single observation at initiation may be subject to biases.

**Methods:**

We used data from the CASCADE (Concerted Action on SeroConversion to AIDS and Death in Europe) multinational cohort collaboration of HIV‐1 seroconverters. We analysed pre‐ and post‐treatment data of patients with seroconversion dates estimated January 2003–March 2014 (*n *=* *7600 for primary analysis) using a statistical model in which the characteristics of recovery in CD4 counts are determined by multiple predictive factors. Secondary analyses were performed incorporating uncertainty in the exact timing of seroconversion to allow more precise estimation of the benefit of very early treatment initiation.

**Results:**

‘True’ CD4 count at cART initiation was the strongest predictor of CD4 count beyond 3 years on cART. Allowing for lack of complete certainty in the date of seroconversion, CD4 recovery was more rapid for patients in whom treatment was initiated within 4 months. For a given CD4 count, higher viral load (VL) at initiation was strongly associated with higher post‐treatment CD4 recovery. For other patient and drug characteristics, associations with recovery were statistically significant but small in magnitude.

**Conclusions:**

CD4 count at cART initiation is the most important factor in predicting post‐treatment recovery, but VL provides substantial additional information. If cART is initiated in the first 4 months following seroconversion, recovery of CD4 counts appears to be more rapid.

## Introduction

Observational studies have consistently found that, for HIV‐1 positive patients initiating combination antiretroviral therapy (cART), higher CD4 cell counts at baseline are associated with higher CD4 count levels after several years of treatment 1, 2, 3, 4, 5, 6. It is also known that early initiation of treatment in HIV‐1 positive patients leads to a large reduction in both AIDS and serious non‐AIDS events 7, 8, leading to the consensus recommendation of immediate initiation of cART 9, 10. However, there is still a clinical motivation to develop a better understanding of factors that predict the characteristics of CD4 recovery, because for some patients viral suppression does not translate into full CD4 recovery 11. Previous studies have been conducted with analyses conditional on a single CD4 count at treatment initiation ignoring any prior observations, which may be subject to biases linked to the inherent measurement error and the fact that the decision to initiate treatment may have been based on the observed value 12.

There is some evidence that initiation of cART close to the date of HIV‐1 infection is associated with a higher level of CD4 recovery than that expected by the baseline CD4 cell count alone. Le *et al*. 13 reported that, among patients with a baseline CD4 count < 500 cells/*μ*L, those who initiated cART within 4 months of their estimated date of infection experienced substantially higher CD4 cell levels over the first 4 years on treatment. Ding *et al*. 14 also reported a more rapid initial increase in CD4 count for patients in whom treatment was initiated within 2 months of diagnosis of a recent infection. However, because investigating this issue requires observation of patients with well estimated date of seroconversion, there is a need for further evidence from large seroconverter cohorts.

Higher viral load (VL) prior to treatment also has been found to be associated with better recovery in CD4 counts from baseline 1, 15, 16. However, we wished to investigate the extent to which this association may be due to the fact that, in people who have recently seroconverted, high VL is a marker for a patient being closer to their true date of seroconversion.

In this study we present results from a novel analysis of the pre‐ and post‐treatment CD4 data of a large dataset of HIV‐1 seroconverters, with the aim of assessing the associations with CD4 response of all available relevant patient and drug regimen characteristics within a single statistical model. We also present an analysis in which uncertainty in the exact date of seroconversion for each patient is taken into account, with the aim of evaluating the potential additional benefit conveyed by very early treatment initiation and the timescale within which this can be obtained. Our modelling approach makes use of all pre‐treatment CD4 count data, accounts for measurement error in these observations, and does not rely on the stratification of continuous predictors of post‐treatment recovery (i.e. grouping into arbitrary subsets). This, in combination with the use of a large dataset of patients with well estimated date of seroconversion, has enabled a more comprehensive analysis of post‐treatment recovery in CD4 counts than has been reported previously in the literature.

## Methods

We fitted combined models 17 of pre‐ and post‐treatment CD4 counts to the full CASCADE (Concerted Action on SeroConversion to AIDS and Death in Europe) within EuroCoord (www.EuroCoord.net) cohort collaboration dataset 18 of seroconverters up until March 2014. Patients who have an interval between last negative and first positive HIV‐1 test < 3 years can be recruited into this cohort collaboration. All cohorts contributing to CASCADE received ethics approval from their individual ethics review boards.

We restricted our analysis to patients with an estimated date of HIV‐1 seroconversion during or after 2003, with this cut‐off chosen to balance the needs for an adequate number of patients and length of follow‐up with the aim for the analysis to reflect relatively modern treatment regimes. Patients who started a regimen of antiretroviral drugs that did not correspond to the definition of cART given below were excluded, as were patients without post‐treatment CD4 count measurements. Those patients without pre‐treatment CD4 counts were not excluded, because the ‘true’ baseline value can still be modelled without this information. cART is defined as a regimen of at least three drugs, with at least two different drug classes (unless abacavir or tenofovir is used in a ‘3N’ regimen with three nucleoside/nucleotide analogue reverse transcriptase inhibitors (NRTI)), and regimens meeting this general definition were further subdivided for analysis. The nonnucleoside reverse transcriptase inhibitor (NNRTI) regimen included regimens with at least one NNRTI and at least two NRTI. The ritonavir‐boosted protease inhibitor (r/PI) regimen included at least one r/PI with at least two NRTI. The integrase strand transfer inhibitor (INSTI) regimen included at least one INSTI with any combination of NRTI, NNRTI and PI. Drug regimens that met the general definition for cART, but not those for the specific NNRTI, r/PI or INSTI regimens, were grouped into an ‘other’ category.

These criteria resulted in a total of 8838 patients from 22 contributing cohorts. We were interested in evaluating the associations between response to cART and multiple patient characteristics and so we also initially excluded patients for whom no VL measurement was recorded within 6 months before the start of cART (*n *=* *910) and those for whom the mode of infection was unknown (*n* = 366). The resulting cohort for analysis included a total of 7600 patients, with 39 225 pre‐treatment and 61 487 post‐treatment CD4 counts.

There was no hepatitis C virus (HCV) test recorded prior to cART in a substantial proportion of patients in the analysis cohort (1309 of 7600) and, as such, we included these patients as a separate grouping in the analysis along with those with a positive (*n *=* *410) or negative (*n *=* *5881) HCV test (based on all available tests: antibody, b‐DNA or RNA). Other patient characteristics included in the analysis were sex, age at treatment initiation and diagnosis of an AIDS‐defining condition prior to treatment.

The analysis was conducted according to drug regimen at initiation of cART, without adjustment of treatment indicators at change to regimen. We censored follow‐up at recorded interruption of cART for more than 1 week, but have not censored follow‐up at change of cART regimen. Analyses were first conducted without censoring due to virological failure. However, we also conducted analyses in which post‐treatment measurements are censored at the observation of any detectable VL beyond 6 months after the initiation of cART in order to provide an estimate of CD4 recovery for individuals who we assumed to have adhered to treatment.

### Statistical analysis

The modelling framework followed that described by Stirrup *et al*. 17, but with an extension to the asymptotic growth curve attributed to Janoshek and Sager 19 to model the shape of post‐treatment recovery in CD4 counts:

$$g\left(t_{\mathrm{post}},u_{i}\right)=\;\phi_{1:i}\;+\;\left(u_{i}-\phi_{1:i}\right)*\exp\left(-\exp\left(\phi_{2:i}\right)*t_{\mathrm{post}}^{D}\right),$$

where *g* is a function of the baseline CD4 count level *u*_*i*_ and time since treatment initiation *t*_post_, *ϕ*_1:*i*_ represents the long‐term maximum average CD4 count, *ϕ*_2:*i*_ relates to the speed of transition to this value from the baseline level and *D* is an additional parameter to be estimated. Both *ϕ*_1:*i*_ and *ϕ*_2:*i*_ are modelled as being dependent on *u*_*i*_ as well as other patient characteristics.

The methodology constitutes an extension of the non‐linear mixed effects framework in which the characteristics of CD4 recovery are modelled as conditional on a latent variable representing the ‘true’ baseline CD4 count at treatment initiation, also including stochastic processes in the models 17, 20. Following previous work 17, the pre‐treatment model comprises a ‘random intercept and slope model’ with a fractional Brownian motion stochastic process included in the variance structure alongside the standard measurement error term. We carried out maximum likelihood estimation using the ADMB software 21. We conducted modelling using the square‐root scale for CD4 counts, but predictions from fitted models were back‐transformed to the original scale. This transformation was found to be appropriate by Taylor and Law 22, and we also present the results of diagnostic checks of residuals from our fitted models in the Appendix S1.

For the initial analysis, we treated the estimated date of seroconversion as fixed, taking the recorded date of seroconversion illness or laboratory evidence of seroconversion (real‐time PCR positivity or antigen positivity with fewer than four bands on Western blot), if observed, or the mid‐point between last negative and first positive HIV‐1 tests if not. For these analyses, we stratified modelling of response to treatment according to estimated timing of treatment initiation from seroconversion (*t*_trt_, in years) as follows: 0 ≤ *t*_trt_ ≤ 0.5, 0.5 < *t*_trt_ ≤ 1.0 or 1.0 < *t*_trt_.

We evaluated improvements in model fit resulting from the addition of potential predictive factors for the characteristics of post‐treatment recovery using likelihood ratio tests. We used flexible natural cubic spline functions to link baseline ‘true’ CD4, VL at treatment initiation and patient age to the characteristics of response to treatment (Section 1.2 in Appendix S1). A model was fitted in which CD4 recovery was also dependent on calendar year of cART initiation: 2003–2006, 2007–2010 or 2011–2014.

We then conducted analyses using a further extension to the statistical model taking into account uncertainty in the exact timing of seroconversion for those patients in whom a `mid‐point’ estimate was used initially 23, 24, incorporating a probability model for pre‐treatment VL observations 25. A scaled beta distribution was used for the true date of infection in each patient, rather than a uniform distribution, for computational stability. For these models, we used a smoothed transition from a typical ‘early treatment response’ to a ‘late treatment response’, allowing evaluation of the time cut‐off for any potential additional benefit (Section 2 in Appendix S1).

## Results

The characteristics of the patients included in the primary analysis are summarized in Table 1. We first describe the results from the primary analysis, with fixed estimates of seroconversion date and without censoring at post‐treatment virological failure.

**Table 1:** Demographic and treatment characteristics of patients included in the primary analysis (*n *=* *7600)

Characteristic	*n* (%) or median (IQR)
Calendar date of SC	26 May 2006 (30 Aug 2004–6 Jul 2008)
SC date estimated by	
SC illness	250 (3.3)
Lab evidence	1490 (19.6)
Mid‐point	5860 (77.1)
Interval between HIV‐1 tests (years)*	0.84 (0.44– 1.5)
Infection group	
MSM	5736 (75.5)
Male heterosexual	722 (9.5)
Male IDU	157 (2.1)
Female heterosexual	936 (12.3)
Female IDU	49 (0.6)
Pre‐cART VL (log_10_(copies/mL))	4.83 (4.25– 5.33)
Age at cART initiation (years)	34.0 (27.8– 41.5)
Pre‐cART AIDS Dx	226 (3.0)
Pre‐cART HCV test	
Positive	410 (5.4)
Negative	5881 (77.4)
Not available	1309 (17.2)
Time from SC to cART (years)	1.40 (0.61–2.72)
0 ≤ *t*_trt_ ≤ 0.5	1613 (21.2)
0.5 < *t*_trt_ ≤ 1.0	1260 (16.6)
1.0 < *t*_trt_	4727 (62.2)
cART regimen	
NNRTI	2989 (39.3)
r/PI	3872 (50.9)
INSTI	438 (5.8)
Other	301 (4.0)
3N	78 (25.9)
Other PI	153 (50.8)
Fusion inhibitor	43 (14.2)
Other classification	27 (9.0)
*n* pre‐cART CD4 counts	4 (1–7)
*n* post‐cART CD4 counts	6 (3–11)
Time to last recorded post‐cART CD4 count (years)†	1.78 (0.72–3.48)

3N, triple nucleoside analogue reverse transcriptase inhibitors; cART, combination antiretroviral therapy; Dx, diagnosis; HCV, hepatitis C virus; IDU, injecting drug user; INSTI, integrase strand transfer inhibitor; IQR, interquartile range; MSM, men who have sex with men; NNRTI, nonnucleoside reverse transcriptase inhibitor; PI, protease inhibitor; r/PI, ritonavir‐boosted PI; SC, seroconversion; *t*_trt_, time from SC to cART (years).

Mid‐point estimates of seroconversion date are used for data shown in this table. *Of those used for mid‐point estimates of SC date. ^†^From date of cART initiation, of those observations included in the analysis.

**Figure 1:**
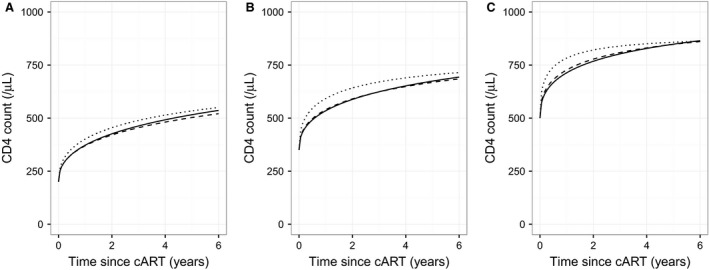
Plots of predicted median recovery in CD4 counts following initiation of combination antiretroviral therapy (cART), from model based on fixed estimated date of seroconversion and without censoring at post‐treatment observation of virological failure, for patients with a ‘true’ baseline value 17 of 200 (a), 350 (b) or 500 (c) cells/*μ*L. Predictions are shown for patients initiating treatment within 6 months of seroconversion (dotted line), patients initiating treatment beyond 6 months but within 1 year (dashed line) and for patients who started treatment beyond 1 year (continuous line). For this plot, all patients are assumed to be men who have sex with men, aged 36 years, with negative test for hepatitis C virus, no prior AIDS diagnosis and starting on a nonnucleoside reverse transcriptase inhibitor (NNRTI) regimen. Viral load prior to treatment is also fixed at the overall median of 4.825 on the log_10_(copies/mL) scale.

**Figure 2:**
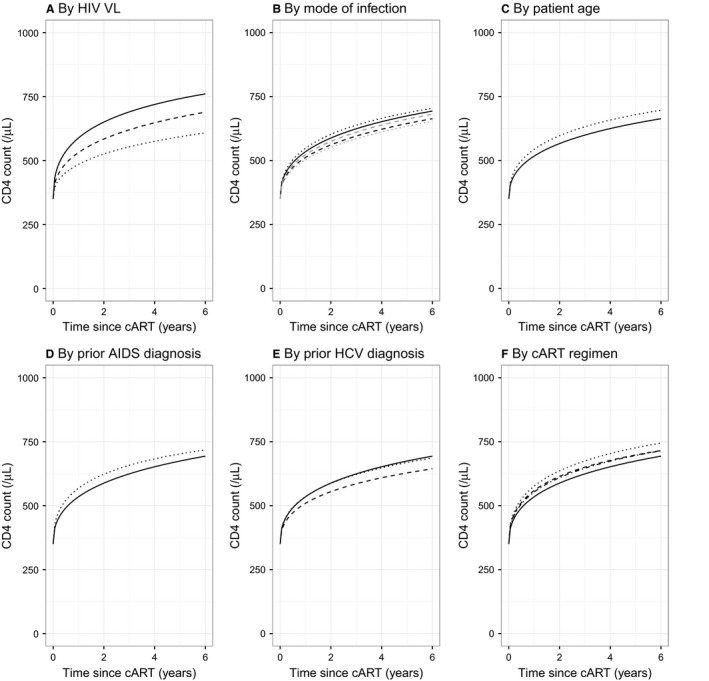
Plots of predicted median recovery in CD4 counts, from model based on fixed estimated date of seroconversion and without censoring at post‐treatment observation of virological failure, for patients with a ‘true’ baseline value (17) of 350 cells/*μ*L according to: (a) viral load (VL) prior to treatment initiation of log_10_(VL) = 2.7 (dotted line), log_10_(VL) = 4.7 (dashed line) or log_10_(VL) = 5.7 (continuous line); (b) sex and infection groups: men who have sex with men (continuous line), male heterosexual (dashed line), male injecting drug user (dotted line); female heterosexual (grey dashed line), female injecting drug user (grey dotted line); (c) patient age at treatment initiation: 20 years (dotted line), 60 years (continuous line); (d) AIDS diagnosis prior to treatment: yes (dotted line), no (continuous line); (e) hepatitis C virus (HCV) status: no test (dotted line), positive test (dashed line), negative test (continuous line); and (f) combination antiretroviral therapy (cART) regimen: integrase strand transfer inhibitor (dotted line), ritonavir‐boosted protease inhibitor (dash‐dot line), other (dashed line), nonnucleoside reverse transcriptase inhibitor (NNRTI) (continuous line). Unless stated otherwise, all patients are assumed to be men who have sex with men, aged 36 years, with negative test for HCV, no prior AIDS diagnosis, baseline log_10_(VL) = 4.825 and starting on a NNRTI regimen at more than 1 year since estimated date of seroconversion.

The use of a Janoshek–Sager curve for post‐treatment CD4 recovery provided a better fit to the data in comparison to a standard asymptotic curve (*P *<* *0.0001). The shape of the fitted curve indicated that, on average, there is a rapid improvement in CD4 count in the 2–3 months following cART initiation with a subsequent gradual increase that continues in most cases for more than 5 years (Fig. 1). We found that the most important factor in predicting the long‐term maximum of CD4 recovery was the baseline CD4 value at treatment initiation (Figs 1 and 2; Table 1.2 in Appendix S1). The group of patients with initiation of treatment within 6 months of estimated date of seroconversion demonstrated a higher speed of recovery (as compared to 6–12 or 12+ months, *P *<* *0.0001), adjusted for CD4 count at treatment initiation, but similar levels beyond around 5 years on cART were predicted by the model (Fig. 1).

Conditional on baseline CD4 level and the time elapsed from seroconversion to treatment, higher VL at treatment initiation was associated with substantially better post‐treatment recovery in CD4 counts (Fig. 2a; illustrative comparisons at 3 years on cART for patient with a CD4 cell count of 350 cells/*μ*L at initiation: 690 cells/*μ*L for log_10_(VL) = 5.7 *vs*. 620 cells/*μ*L for log_10_(VL) = 4.7); this provides evidence that, for a given baseline CD4 level, baseline VL is positively linked to CD4 recovery following cART initiation independent of the time since infection. Once the baseline CD4 and VL at treatment initiation and the time from seroconversion to treatment had been included in the model, estimated effect sizes for additional patient and drug characteristics were small. However, all were statistically significant, with *P *<* *0.005, a consequence of the large sample size, and so we present results that relate to the full model containing all factors. CD4 recovery is predicted to be slightly worse for male patients infected through sex between men and women (Fig. 2b; 596 cells/*μ*L at 3 years *vs*. 624 cells/*μ*L for men who have sex with men), and recovery is predicted to be better on average in younger patients (Fig. 2c; 599 cells/*μ*L at 3 years for 60‐year‐old *vs*. 632 cells/*μ*L for 20‐year‐old). An AIDS diagnosis prior to treatment initiation was associated with slightly better recovery (Fig. 2d; 658 cells/*μ*L at 3 years with previous AIDS diagnosis *vs*. 624 cells/*μ*L without) and a positive HCV test prior to treatment initiation was associated with slightly worse recovery (Fig. 2e; 585 cells/*μ*L at 3 years with positive HCV test *vs*. 624 cells/*μ*L with negative test). Of the cART regimens at treatment initiation, patients initiating cART with an INSTI showed improved CD4 recovery (Fig. 2f; 675 cells/*μ*L at 3 years for INSTI *vs*. 624 cells/*μ*L for NNRTI).

**Figure 3:**
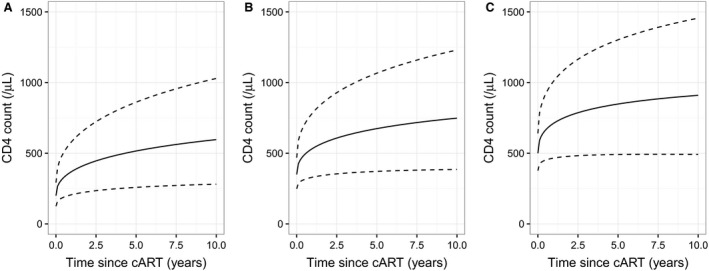
Plots of predicted median (continuous line) and 5th and 95th percentiles (dashed line) for recovery in CD4 counts following initiation of combination antiretroviral therapy (cART), from model based on fixed estimated date of seroconversion and without censoring at post‐treatment observation of virological failure, for patients with a ‘true’ baseline value 17 of 200 (a), 350 (b) or 500 (c) cells/*μ*L. For this plot, all patients are assumed to be men who have sex with men, aged 36 years, with negative test for hepatitis C virus, no prior AIDS diagnosis and starting on a nonnucleoside reverse transcriptase inhibitor (NNRTI) regimen beyond 1 year from seroconversion. Viral load prior to treatment is also fixed at the overall median of 4.825 on the log_10_(copies/mL) scale. In these graphs, predictions are extrapolated to the full range of the post‐treatment data.

The residual unexplained variation between patients in their post‐treatment recovery in CD4 counts was substantial (Fig. 3). None of the findings described were substantively altered by the reanalysis of a dataset in which post‐treatment CD4 counts were censored following the observation of a detectable VL beyond 6 months from cART initiation (Sections 1.3 and 1.4 in Appendix S1). The model including calendar period for cART initiation showed a significant improvement in fit (*P *=* *0.007), but with minimal changes in the parameter estimates associated with other variables.

When uncertainty in precise date of seroconversion was taken into account, we found that there was a more rapid CD4 recovery when treatment was initiated within 4 months of seroconversion (Section 2.5 in Appendix S1). The findings relating to patient and drug regimen characteristics were very similar to those described for the models assuming a fixed estimate of seroconversion date. For the models incorporating uncertainty in exact seroconversion date, the finding that higher VL was associated with substantially better post‐treatment recovery in CD4 counts persisted, conditional on baseline CD4 level and the time elapsed from seroconversion to treatment. It should be noted that VL levels for each individual were found to be negatively correlated with both the intercept ($\hat{r}$ = −0.28) and slope ($\hat{r}$ = −0.50) of pre‐treatment CD4 counts (i.e. higher pre‐treatment VL was associated with lower CD4 count at seroconversion and with a steeper decline), which is important given that the positive association with CD4 recovery is conditional on the baseline CD4 count at treatment initiation.

## Discussion

We found that the most important predictors of recovery in CD4 counts in patients with well‐estimated dates of seroconversion following the initiation of cART are the CD4 count and VL at time of treatment initiation. The strong positive association of CD4 count at treatment initiation with the long‐term maximum of post‐treatment recovery was expected given the findings of previous research on this topic 1, 2, 3, 4, 5, 6.

Higher VL at treatment initiation was found to predict more rapid recovery in CD4 counts, for a given baseline CD4 count and time elapsed from seroconversion, with sustained higher values over the time frame considered. This finding persisted when uncertainty in the exact date of seroconversion was taken into account. There is some evidence that higher plasma VL levels are associated with sequestration of CD4 cells in lymphoid tissue 26, 27, and it has been suggested that this is associated with a more rapid initial increase in circulating CD4 cells following the initiation of cART 28, 29; this may also imply that, in cases with high pre‐treatment VL, the baseline level of circulating CD4 cells provides a slightly pessimistic indicator of the underlying state of the immune system at treatment initiation. An alternative explanation for the fact that low pre‐treatment VL predicts lower CD4 recovery is that this may reflect previous exposure to ART that has not been recorded. However, we think that this is not likely to be the main cause of this finding in our analysis because all patients were under continuous observation from the time of HIV‐1 diagnosis.

Initiation of cART close to the date of seroconversion was also found to be associated with a more rapid increase in CD4 counts for any given baseline CD4 count and VL level. This finding is consistent with previous research on this topic in smaller cohorts 13, 14. In our primary analysis, assuming fixed estimates of seroconversion date for each patient, a more rapid response to treatment was observed when cART was initiated within 6 months, but not when it was initiated at 6–12 months. In the analysis in which uncertainty in the date of seroconversion was taken into account, we found that an additional benefit in CD4 response over the first 3 years of treatment could be observed when cART was initiated within 4 months of seroconversion, matching the finding of Le *et al*. 13. Because CD4 counts decline over time in untreated patients, these results imply that substantially better CD4 recovery would be expected for patients that initiate treatment close to their date of seroconversion. We note that these analyses are only possible due to the information provided in the CASCADE dataset regarding the dates of negative and positive HIV tests for each patient.

A previous study by Mussini *et al*. 28 analysing an earlier CASCADE dataset found that, controlling for both baseline CD4 count and VL, steeper pre‐treatment declines in CD4 count were associated with more rapid recovery once treatment was initiated. Similarly Jarrin *et al*. 29, again using CASCADE data, found that initial recovery at 1 month after treatment initiation was increased in ‘rapid progressors’ (patients with at least one CD4 cell count < 200 cells/*μ*L within 12 months of seroconversion). It would have been interesting to have also tested for an independent effect of rate of pre‐treatment CD4 decline in the present analysis, but this was not attempted following the failure to fit such models in our pilot investigation 17.

The other patient and drug regimen characteristics that were included in our analysis only showed small to moderate associations with post‐treatment recovery in CD4 counts. Because this is an analysis of an observational dataset, observed differences that are small in magnitude need to be interpreted with caution. Increasing patient age at date of treatment initiation was found to be associated with a moderate reduction in CD4 recovery conditional on the baseline value, which is consistent with previous research 1, 16, 30. Pre‐treatment diagnosis of HCV also was found to be associated with a moderate reduction in recovery; although statistically significant differences in CD4 recovery have not always been found for cases of HCV 16, 30, this is a finding that has been observed in other studies 31, 32, 33. A limitation of our analysis is that we did not account for the potential for treatment and cure of HCV prior to the initiation of cART, although the latter is unlikely given that the seroconversions largely pre‐dated the use of direct acting antivirals. Another limitation is that the presence of a chronic HCV infection prior to cART initiation was not determined in some patients included in the analysis.

The finding that a pre‐treatment AIDS diagnosis was associated with an improvement in the post‐treatment recovery could be considered surprising, but it should be noted that the effect size was small, that this is conditional on baseline CD4 count and that we did not distinguish between the severity of the various conditions included. It is possible that the difference observed could be explained by greater sequestration of CD4 cells in the lymphoid tissue in such cases, leading to a greater increase on initiation of cART, as has been suggested for cases with higher pre‐treatment VL 26, 27. No substantial difference in response was present for the analysis in which uncertainty in the true date of seroconversion was taken into account and censoring was applied at post‐cART virological failure (Section 2.5 in Appendix S1); as such, it is possible that the observed effect could be due to better adherence to treatment in those patients with an AIDS diagnosis.

Comparison of recovery by sex and infection group in the present analysis is hampered by the fact that the sample size for some subgroups is small. Recovery was observed to be slightly worse on average amongst heterosexual men, which has not been reported previously. However, it is possible that this difference could be due to correlated factors such as ethnicity and associated differences in socioeconomic circumstances, for which data were only available in a minority of patients in this cohort. Some previous studies have observed better post‐treatment CD4 recovery in women 1, 30, but we did not find such an association.

Of the classifications of drug regimens included in the analysis, use of an INSTI regimen at treatment initiation was found to be associated with a moderate improvement in post‐treatment recovery in CD4 relative to the NNRTI regimen. These findings warrant further investigation, but are not conclusive on their own given the potential for residual confounding and the moderate effect sizes found. There is evidence that the use of INSTI regimens is associated with more rapid viral suppression, but the evidence regarding potential differences in CD4 recovery is less clear 34, 35, 36, 37. A network meta‐analysis found a slightly larger CD4 cell count (≈20 cells/*μ*L) at 48 weeks after initiation of ART regimens containing an INSTI 38.

No substantial differences were observed when the analysis was repeated for a processed dataset with censoring of post‐treatment CD4 counts following the observation of a detectable VL beyond 6 months from initiation of cART. This is surprising given that the observation of a detectable VL on cART could be taken to indicate poor adherence. The fact that no difference was observed when censoring was applied could be explained by sufficiently high levels of drug adherence within the studied cohort or by effective recording of treatment interruptions. The level of unexplained residual variation in CD4 recovery on cART remained high in these analyses, indicating that the clinical factors included in our models do not allow accurate prediction of those patients who will go on to show suboptimal CD4 recovery on treatment despite successful viral suppression.

In this study we provide a comprehensive analysis of factors that predict recovery in CD4 cell counts following initiation of cART in HIV‐1 patients. Our findings are largely consistent with those reported previously in the literature, but we provide further information regarding the timescale for the potential additional benefit conveyed by early treatment initiation, after adjusting for baseline CD4 count, and also demonstrate the relative importance of different factors. The findings also provide further supporting evidence for the initiation of cART soon after HIV diagnosis, as now recommended in World Health Organization guidelines 39. We found a large amount of unexplained variance in post‐treatment CD4 cell counts, indicating that further work is required to achieve the goal of accurate prediction of suboptimal response to cART.

## Supporting information


**Appendix S1** Document including in‐depth description of the statistical methodology, further details of the fitted models and some further discussion of the results.

## Appendix

CASCADE Steering Committee: Julia Del Amo (Chair), Laurence Meyer (Vice Chair), Heiner C. Bucher, Geneviève Chêne, Osamah Hamouda, Deenan Pillay, Maria Prins, Magda Rosinska, Caroline Sabin, Giota Touloumi.

CASCADE Co‐ordinating Centre: Kholoud Porter (Project Leader), Ashley Olson, Andrea Cartier, Lorraine Fradette, Sarah Walker, Abdel Babiker.

CASCADE Clinical Advisory Board: Heiner C. Bucher, Andrea De Luca, Martin Fisher, Roberto Muga.

CASCADE Collaborators: Australia PHAEDRA cohort (Tony Kelleher, David Cooper, Pat Grey, Robert Finlayson, Mark Bloch), Sydney AIDS Prospective Study and Sydney Primary HIV Infection cohort (Tony Kelleher, Tim Ramacciotti, Linda Gelgor, David Cooper, Don Smith); Austria Austrian HIV Cohort Study (Robert Zangerle); Canada South Alberta clinic (John Gill); Estonia Tartu Ülikool (Irja Lutsar); France ANRS CO3 Aquitaine cohort (Geneviève Chêne, Francois Dabis, Rodolphe Thiebaut), ANRS CO4 French Hospital Database (Dominique Costagliola, Marguerite Guiguet), Lyon Primary Infection cohort (Philippe Vanhems), French ANRS CO6 PRIMO cohort (Marie‐Laure Chaix, Jade Ghosn), ANRS CO2 SEROCO cohort (Laurence Meyer, Faroudy Boufassa); Germany German HIV‐1 seroconverter cohort (Osamah Hamouda, Karolin Meixenberger, Norbert Bannert, Barbara Bartmeyer); Greece AMACS (Anastasia Antoniadou, Georgios Chrysos, Georgios L. Daikos); Greek Haemophilia cohort (Giota Touloumi, Nikos Pantazis, Olga Katsarou); Italy Italian Seroconversion Study (Giovanni Rezza, Maria Dorrucci), ICONA cohort (Antonella d'Arminio Monforte, Andrea De Luca); Netherlands Amsterdam Cohort Studies among homosexual men and drug users (Maria Prins, Ronald Geskus, Jannie van der Helm, Hanneke Schuitemaker); Norway Oslo and Ulleval Hospital cohorts (Mette Sannes, Oddbjorn Brubakk, Anne‐Marte Bakken Kran); Poland National Institute of Hygiene (Magdalena Rosinska); Spain Badalona IDU hospital cohort (Roberto Muga, Jordi Tor), Barcelona IDU Cohort (Patricia Garcia de Olalla, Joan Cayla), CoRIS‐scv (Julia del Amo, Santiago Moreno, Susana Monge); Madrid cohort (Julia Del Amo, Jorge del Romero), Valencia IDU cohort (Santiago Pérez‐Hoyos); Sweden Swedish InfCare HIV Cohort, Sweden (Anders Sönnerborg); Switzerland Swiss HIV Cohort Study (Heiner C. Bucher, Huldrych Günthard, Alexandra Scherrer); Ukraine Perinatal Prevention of AIDS Initiative (Ruslan Malyuta); United Kingdom Public Health England (Gary Murphy), UK Register of HIV Seroconverters (Kholoud Porter, Anne Johnson, Andrew Phillips, Abdel Babiker), University College London (Deenan Pillay); African cohorts: Genital Shedding Study (US: Charles Morrison; Family Health International, Robert Salata, Case Western Reserve University, Uganda: Roy Mugerwa, Makerere University, Zimbabwe: Tsungai Chipato, University of Zimbabwe); International AIDS Vaccine Initiative (IAVI) Early Infections Cohort (Kenya, Rwanda, South Africa, Uganda, Zambia: Matt A. Price, IAVI, USA; Jill Gilmour, IAVI, UK; Anatoli Kamali, IAVI, Kenya; Etienne Karita, Projet San Francisco, Rwanda).

EuroCoord Executive Board: Fiona Burns, University College London, UK; Geneviève Chêne, University of Bordeaux, France; Dominique Costagliola (Scientific Coordinator), Institut National de la Santé et de la Recherche Médicale, France; Carlo Giaquinto, Fondazione PENTA, Italy; Jesper Grarup, Region Hovedstaden, Denmark; Ole Kirk, Region Hovedstaden, Denmark; Laurence Meyer, Institut National de la Santé et de la Recherche Médicale, France; Heather Bailey, University College London, UK; Alain Volny Anne, European AIDS Treatment Group, France; Alex Panteleev, St. Petersburg City AIDS Centre, Russian Federation; Andrew Phillips, University College London, UK; Kholoud Porter, University College London, UK; Claire Thorne, University College London, UK.

EuroCoord Council of Partners: Jean‐Pierre Aboulker, Institut National de la Santé et de la Recherche Médicale, France; Jan Albert, Karolinska Institute, Sweden; Silvia Asandi, Romanian Angel Appeal Foundation, Romania; Geneviève Chêne, University of Bordeaux, France; Dominique Costagliola (chair), INSERM, France; Antonella d'Arminio Monforte, ICoNA Foundation, Italy; Stéphane De Wit, St. Pierre University Hospital, Belgium; Peter Reiss, Stichting HIV Monitoring, Netherlands; Julia Del Amo, Instituto de Salud Carlos III, Spain; José Gatell, Fundació Privada Clínic per a la Recerca Bíomèdica, Spain; Carlo Giaquinto, Fondazione PENTA, Italy; Osamah Hamouda, Robert Koch Institut, Germany; Igor Karpov, University of Minsk, Belarus; Bruno Ledergerber, University of Zurich, Switzerland; Jens Lundgren, Region Hovedstaden, Denmark; Ruslan Malyuta, Perinatal Prevention of AIDS Initiative, Ukraine; Claus Møller, Cadpeople A/S, Denmark; Kholoud Porter, University College London, United Kingdom; Maria Prins, Academic Medical Centre, Netherlands; Aza Rakhmanova, St. Petersburg City AIDS Centre, Russian Federation; Jürgen Rockstroh, University of Bonn, Germany; Magda Rosinska, National Institute of Public Health, National Institute of Hygiene, Poland; Manjinder Sandhu, Genome Research Limited; Claire Thorne, University College London, UK; Giota Touloumi, National and Kapodistrian University of Athens, Greece; Alain Volny Anne, European AIDS Treatment Group, France.

EuroCoord External Advisory Board: David Cooper, University of New South Wales, Australia; Nikos Dedes, Positive Voice, Greece; Kevin Fenton, Public Health England, USA; David Pizzuti, Gilead Sciences, USA; Marco Vitoria, World Health Organization, Switzerland.

EuroCoord Secretariat: Silvia Faggion, Fondazione PENTA, Italy; Lorraine Fradette, University College London, UK; Richard Frost, University College London, UK; Andrea Cartier, University College London, UK; Dorthe Raben, Region Hovedstaden, Denmark; Christine Schwimmer, University of Bordeaux, France; Martin Scott, UCL European Research & Innovation Office, UK.
