# Supplementary material for: Predictors of CD4 cell recovery following initiation of antiretroviral therapy among HIV‐1 positive patients with well‐estimated dates of seroconversion
Source: HIV Med. 2017 Dec 1;19(3):184–94. doi: 10.1111/hiv.12567 (PMC5836945; doi:10.1111/hiv.12567)
Supplement: Supplementary file 1 — Appendix S1 Document including in‐depth description of the statistical methodology, further details of the fitted models and some further discussion of the results. [file HIV-19-184-s001.pdf]

Appendix to: Predictors of CD4 cell recovery following initiation of antiretroviral therapy among HIV-1 positive patients with well-estimated dates of seroconversion

Stirrup *et al.*

September 18, 2017

1 Primary analysis: application of combined model to CASCADE dataset

1.1 Dataset and estimation

We apply the modelling framework developed by Stirrup *et al.*[1] to the full CASCADE dataset of seroconverters. We restrict our analysis to patients with an estimated date of HIV seroconversion during or after 2003, and patients who started a suboptimal regimen of antiretroviral drugs prior to cART were excluded as were patients without at least one post-treatment CD4 count recorded. Data were included up to March 2014. CD4 counts are modelled on a square-root scale, with back-transformation of predictions for hypothetical patients.

The primary analysis relates to models fitted with a latent variable for each patient only for the 'true' baseline CD4 value (on the square-root scale) at initiation of cART, with maximum likelihood estimation carried out using 10-point adaptive Gauss–Hermite quadrature. Maximum likelihood estimation was carried out using the random effects mode of the ADMB software, run on the UCL Legion High Performance Computing Facility.

1.2 Pre-treatment model structure

The pre-treatment model for CD4 counts comprised a 'random intercept and slope' model with a fractional Brownian motion stochastic process component in addition to the independent random error term for each measurement occasion. This matches the model structure for $Model_1$–$Model_5$ as in Stirrup *et al.*[1]. The conditional distribution for the latent variable representing baseline CD4 count was also constructed as described in Stirrup *et al.*[1].

1.3 Post-treatment model structure and hypothesis tests

Given the much larger cohort of patients in this analysis in comparison to that presented previously[1], we allow greater flexibility in the shape of the asymptotic recovery curve. We use an extension to the asymptotic growth curve attributed to Janoshek and Sager[2–4]:

$$g\left(t_{post}, u_i^+\right) = \phi_{1:i} + \left(u_i^+ - \phi_{1:i}\right)\exp\left(-\exp\left(\phi_{2:i}\right) t_{post}^D\right).$$

This function matches that given in Stirrup *et al.*[1] with an additional power transformation of the post-treatment time variable t_{post} by exponent D, a parameter to be estimated with value $D > 0$. For values of $D > 1$ the growth curve is sigmoidal, for

$D = 1$ growth follows a standard asymptotic curve and for $D < 1$ growth is more rapid at time points closer to zero. The $\phi_{1:i}$ and $\phi_{2:i}$ terms still reflect long-term maximum and speed of recovery, respectively, but parameter estimates may not be straightforward to interpret directly if recovery does not reach the modelled asymptotic maximum within the time-frame under consideration (i.e. if substantial recovery in CD4 counts is still ongoing beyond around 5 years after initiation of cART).

Given the accurate approximation to the marginal log-likelihood for each fitted model in the primary analysis (with a latent variable term only included for the 'true' baseline), statistical hypothesis tests for comparison of nested models are carried out using generalised likelihood-ratio tests. We initially fit a model in which the post-treatment recovery in CD4 count follows a standard asymptotic curve, for which the long-term maximum and speed of response are each linearly dependent on the 'true' baseline CD4 value (u^+) but not on any other patient or treatment characteristics. We then test whether the Janoshek and Sager curve provides a better fit to the data. Following Stirrup *et al.*[1], we subsequently stratify the functions that specify the dependence of $\phi_{1:i}$ and $\phi_{2:i}$ on u_i^+ according to whether treatment was initiated within 6 months, beyond 6 months and within 1 year or beyond 1 year from the estimated date of seroconversion.

We consider whether VL before treatment initiation is predictive of the speed of recovery in CD4 counts or long-term maximum; VL (in copies/mL) is transformed to the $\log_{10}$ scale and used to generate a natural cubic spline basis with boundary and internal knots at $(3, 4, 4.7, 5, 6)$, no intercept is included (as this would not be identifiable) and the basis is centred at 4.7 (i.e. $\log_{10}(50\,000)$), which results in four model parameters relating to $\phi_{1:i}$ and an additional four relating to $\phi_{2:i}$. Sets of parameters relating to groupings of patients determined by sex and mode of infection are then added to the model; we have combined these characteristics into a single step in model development because of the inherent dependence between sex and mode of infection (i.e. the majority of the cohort are men who have sex with men, but there is not an equivalent group of women) and the potential for sex differences to vary according to whether the patient is an injecting drug user (IDU). Men who have sex with men were treated as the reference group ($n = 5736$), with parameters added to the models for $\phi_{1:i}$ and $\phi_{2:i}$ for heterosexual men and women ($n = 722$ and $n = 936$, respectively) and male and female IDUs ($n = 157$ and $n = 49$, respectively).

Patient age at treatment (in years) is then added to the models for $\phi_{1:i}$ and $\phi_{2:i}$ using a natural cubic spline basis and knots at $(25, 31, 36, 41, 51)$, approximately equivalent to the 10th, 30th, 50th, 70th and 90th centiles. As for VL there is no intercept, and so four parameters are added to the model for both $\phi_{1:i}$ and $\phi_{2:i}$, and the basis is centred at 36 years. Parameters relating to the diagnosis of an AIDS-defining illness prior to initiation of cART ($n = 226$) are then added, followed by parameters linked

to either a positive test for HCV ($n = 410$) or no record of a test for HCV ($n = 1309$) prior to cART. The predictive value of cART regimen classification at initiation was then assessed, with patients grouped with NNRTI regimen as reference ($n = 2989$), and parameters added relating to ritonavir-boosted PI ($n = 3872$), INSTI ($n = 438$) or other treatment regimens ($n = 301$).

Variables were added in perceived order of importance given a reading of previous literature on this topic. However, the drug regimen variable was added last so that the effect would be estimated conditional on all baseline patient characteristics, given that this is the one variable that could be altered in a clinical trial. We also note that as results are presented for the model with all variables included, the order of variable addition is not likely to have had any effect on the findings reported.

After the addition of the specified patient and treatment characteristics to the model, the functions linking (square-root) baseline CD4 value and the speed and long-term maximum of recovery were generalised (from a linear relationship) using a natural cubic spline basis with knots at 15.5, 17.5, 19.5 and 22 (stratified by time from estimated date of seroconversion to treatment initiation, as in Stirrup *et al.*[1]). This was done after the addition of the patient characteristics to the model because the combination of a large number of additional parameters (12) and the use of natural cubic splines applied to a latent variable term increased the required computation time to fit the model up to a level that was close to the maximum available (72 hours).

1.4 Results without censoring due to virological failure

The models fitted to the full CASCADE dataset (without censoring related to VL) are summarised in Table 1.1. Generalising the baseline model (Mod_1) so that CD4 recovery followed a Janoshek–Sager curve (Mod_2) led to a highly significant improvement in model fit ($2\Delta\ell 2510$ for 1 parameter, $P < 0.0001$), and so this extension to the model was maintained. As was found in Stirrup *et al.*[1], stratifying the functions linking baseline CD4 to recovery by the time elapsed from estimated date of seroconversion to treatment initiation also led to a highly significant improvement in model fit (Mod_3; $2\Delta\ell 680$ for 8 parameters; $P < 0.0001$). A further highly significant improvement in model fit was found when VL prior to treatment initiation was added as a predictor (Mod_4; $2\Delta\ell 624$ for 8 parameters; $P < 0.0001$). Adding each of the remaining patient and drug regimen characteristics to the model as predictors (Mod_5–Mod_9) led to statistically significant improvements in model fit (with $P < 0.0001$ in all cases, other than for 'age' for which $P = 0.0011$, and corresponding reductions in AIC); however, the improvement in log-likelihood for each of these models was modest relative to the size of the dataset under investigation, and no further improvements in BIC were seen. Similarly, the use of natural cubic splines to create

more flexible link functions between baseline CD4 and the nature of post-treatment recovery led to a statistically significant improvement in model fit (Mod_{10} vs Mod_9; $2\Delta\ell 68$ for 12 parameters; $P < 0.0001$), but not a reduction in BIC.

Table 1.1. Summary of fitted combined models for CD4 cell counts before and after the initiation of combination antiretroviral therapy (cART) in patients from the CASCADE cohort. All models shown are nested within that described in the row below.

Model	Predictors	Curve	n_{pars}	ℓ	AIC	BIC	$2\Delta\ell$
Mod_1	Linear-u	Asym.	15	-247 019	494 068	494 211	NA
Mod_2	Linear-u	JS	16	-245 764	491 560	491 712	2 510
Mod_3	As above + trt-time grp	JS	24	-245 424	490 896	491 124	680
Mod_4	As above + baseline VL	JS	32	-245 112	490 288	490 593*	624
Mod_5	As above + gender/inf grp	JS	40	-245 096	490 272	490 653	32
Mod_6	As above + age	JS	48	-245 083	490 262	490 719	26
Mod_7	As above + AIDS Dx	JS	50	-245 070	490 240	490 716	26
Mod_8	As above + HCV Dx	JS	54	-245 056	490 220	490 734	28
Mod_9	As above + trt class	JS	60	-245 029	490 178	490 749	54
Mod_{10}	As above + NCS-u	JS	72	-244 995	490 134*	490 819	68

The 'Predictors' field lists variables included in the functions to determine both long-term maximum (ϕ_1) and speed of recovery (ϕ_2), and 'Curve' gives shape of expected recovery following cART. 'trt-time grp' denotes stratification of functions for long-term maximum and speed of recovery in terms of baseline CD4 at treatment initiation. *Lowest value of AIC/BIC for set of models. '$2\Delta\ell$' denotes differences in $2\times$log-likelihood in comparison to model described in the row above in each case. AIC, Akaike information criterion; AIDS, acquired immune deficiency syndrome; Asym., asymptotic; BIC, Bayesian information criterion; Dx, diagnosis prior to cART; grp, group; HCV, hepatitis C virus; inf, mode of infection; JS, Janoshek–Sager; ℓ, log-likelihood of model fit; NA, not applicable; NCS, natural cubic spline; n_{pars}, number of parameters in model; trt, treatment; VL, viral load.

Although it seems therefore that Mod_4 might provide a more parsimonious model for response to treatment, here and in the main paper we further investigate the implications of the fitted Mod_{10} to evaluate the role of patient and drug regimen characteristics to predict response to cART. We wished to estimate associations with CD4 recovery for all potentially relevant variables, and we feel that this is justified by the fact that a large sample size was used relative to the number of parameters estimated and that each expansion of the model from Mod_4 to Mod_{10} was associated with a P-value of less than 0.0001, other than that for age for which the P-value was 0.0011. Although the associations found were not large in magnitude, it is not likely that the variables assessed have zero true association with CD4 recovery.

Parameter estimates for Mod_{10} are given in Table 1.2. Direct interpretation of the parameter estimates is complicated for many of the patient characteristics by the fact that the sign (i.e. $+/-$) of the effect on long-term maximum CD4 is opposite for that on speed of recovery, and so it is not immediately obvious whether or not an associated benefit is predicted. This problem is compounded by the fact that the D pa-

rameter for the Janoshek–Sager curve was estimated to be less than one ($\hat{D}$=0.42 for Mod_{10}), indicating a rapid early response to treatment but with a very gradual later response; this has the effect that the modelled long-term maximum for any given patient is not attained within the time-frame for the available data for this analysis, and so the parameters relating to 'long-term maximum' and 'speed of recovery' cannot be interpreted in isolation. As such, evaluation of the fitted model here and in the main paper is focused on generating and comparing predicted recovery curves for hypothetical patients. However, we start by inspecting the fitted natural cubic spline functions for baseline CD4, VL prior to treatment initiation and patient age.

The link functions for ϕ_1 and ϕ_2 in terms of baseline CD4 (u_i^+), and stratified by elapsed time from estimated date of seroconversion to initiation of cART fitted in Mod_{10} are shown in Figure 1.1. As found in the more limited analysis in Stirrup *et al.*[1], both the long-term maximum and speed of recovery were positively associated with the baseline 'true' CD4 count, and those patients that initiated treatment within 6 months of seroconversion were found to show a more rapid recovery for a given baseline CD4 count. The effect of pre-treatment VL on ϕ_1 and ϕ_2 is plotted in Figure 1.2, VL does not appear to predict the long-term maximum CD4 count after treatment initiation, but higher than average VL values do seem to predict a substantially higher speed of recovery. Patient age at treatment initiation was estimated to have little effect on long-term maximum CD4, and greater age was found to be associated with a small reduction in the speed of recovery (Figure 1.3).

The predicted median recovery in CD4 counts following initiation of cART for a series of hypothetical patients is presented in Figures 1.4 and 1.5. In Figure 1.4 predictions are shown according to 'true' baseline CD4 and time elapsed from estimated date of seroconversion to treatment initiation, again demonstrating the link between baseline CD4 and long-term maximum. The plots also demonstrate that the use of the Janoshek–Sager curve results in a model that predicts (on average) gradual increases in CD4 beyond 5 years from the initiation of cART, even amongst those patients with a high baseline value. The gain in speed of recovery associated with early initiation of cART appears to be only moderate, but high VL prior to treatment is also strongly predictive of a rapid response as shown in Figure 1.5a. VL shows a peak close to the date of seroconversion (e.g. Pantazis *et al.*[5]), and so high VL measurements might be acting as a marker that any given patient is close to their date of seroconversion. The CASCADE dataset includes patients with up to 3 years between their last negative and first positive test for HIV, and so this might be the case even for those patients assigned to the group with greater than 1 year between estimated date of seroconversion and treatment initiation. This is further investigated in Section 2 of this Appendix.

For the remaining patient and treatment characteristics the estimated effect sizes

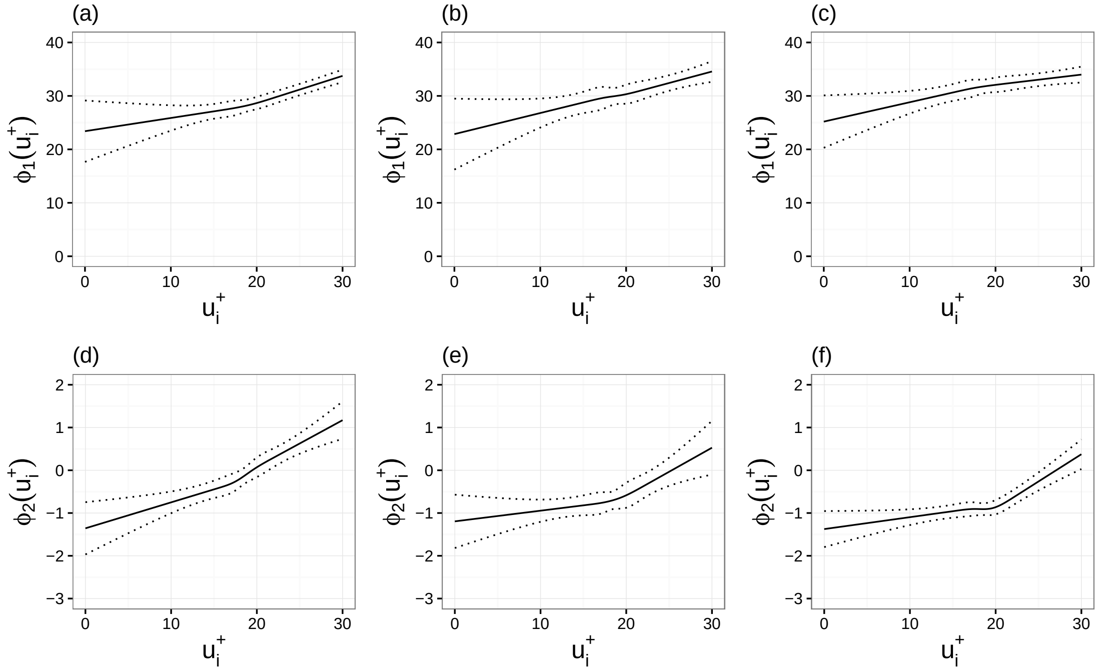

Figure 1.1. Plots of $\phi_1\left(u_i^+\right)$ (a–c, relating to long-term maximum) and $\phi_2\left(u_i^+\right)$ (d–f, relating to speed of response) for Mod_{10}. Graphs on the left of each row (a,d) show the fitted functions for patients initiating treatment within 6 months of seroconversion, those in the centre (b,e) show the functions for patients initiating treatment beyond 6 months but within 1 year and those on the right (c,f) show the functions for patients who started treatment beyond 1 year. Pointwise 95 % confidence intervals for the functions are shown (⋯⋯).

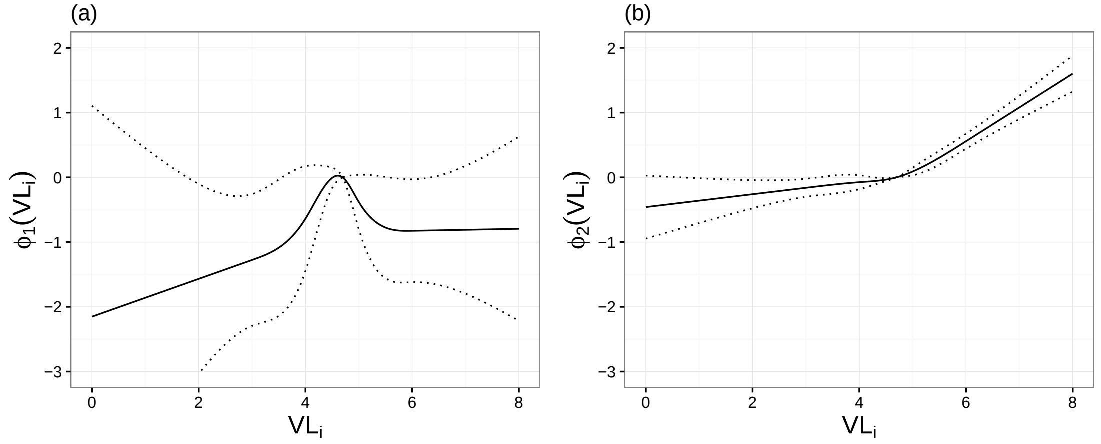

Figure 1.2. Plots of effect on ϕ_1 (a, relating to long-term maximum) and ϕ_2 (b, relating to speed of response) of pre-treatment viral load (VL, expressed using $\log_{10}$ scale on x-axis) as estimated in Mod_{10}. Pointwise 95 % confidence intervals for the functions are shown (⋯⋯). The model is parameterised such that the effect at $\log_{10}$(VL)=4.7 is zero.

were only moderate (Figure 1.5), which makes interpretation difficult given the potential for residual unmeasured confounding factors. Recovery is predicted to be slightly worse for male heterosexuals or female IDUs, but the sample size in the latter group was very small and the 95 CIs of parameter estimates for the effect on ϕ_1

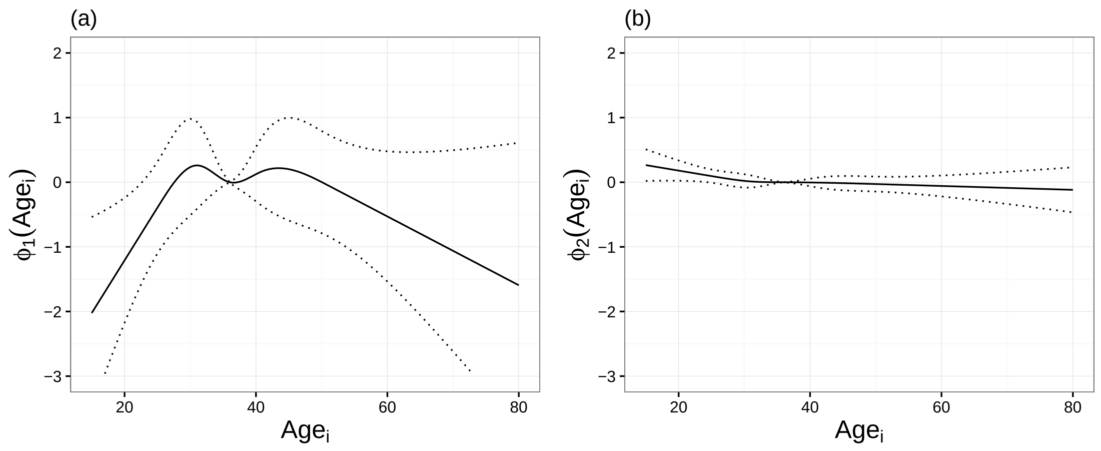

Figure 1.3. Plots of effect on ϕ_1 (a, relating to long-term maximum) and ϕ_2 (b, relating to speed of response) of patient age at treatment initiation as estimated in Mod_{10}. Pointwise 95 % confidence intervals for the functions are shown (······).The model is parameterised such that the effect at 36 years is zero.

and ϕ_2 both included zero. As also demonstrated in Figure 1.3, recovery is predicted to be better on average in younger patients. A surprising finding is that an AIDS diagnosis prior to treatment initiation was associated with slightly better recovery, although the sample size of such patients was small. A positive HCV test prior to treatment initiation was associated with slightly worse recovery. Of the cART regimens, the INSTI category was associated with improved recovery, with the 'other' and ritonavir-boosted PI categories showing the next best performance. However, it is possible that the use of newer drugs is associated with confounding factors such as earlier treatment initiation (as this is only partially controlled for in the current model), and so caution is required in the interpretation of this finding.

The estimates of variance parameters relating to unexplained variation in post-treatment CD4 recovery were large, representing clinically meaningful differences in response to treatment that could not be attributed to the combination of patient and drug characteristics included in the model. This can be seen in both the estimated variance of the random effect term relating to asymptotic maximum ($\hat{P} = 9.9$) and to a lesser extent in the parameters relating to the post-treatment fractional Brownian motion process ($\hat{\kappa}_{post} = 4.6$ and $\hat{H}_{post} = 0.25$). The residual variation is also illustrated in Figure 1.6, in which the 5th and 95th centiles of post-treatment CD4 counts are plotted for hypothetical patients in addition to the median.

1.5 Results with censoring due to virological failure

Due to the requirement for at least one post-treatment CD4 count, censoring at post-treatment virological failure led to a slightly smaller total number of 7543 patients for analysis, with 39 013 pre-treatment and 44 376 post-treatment CD4 count observa-

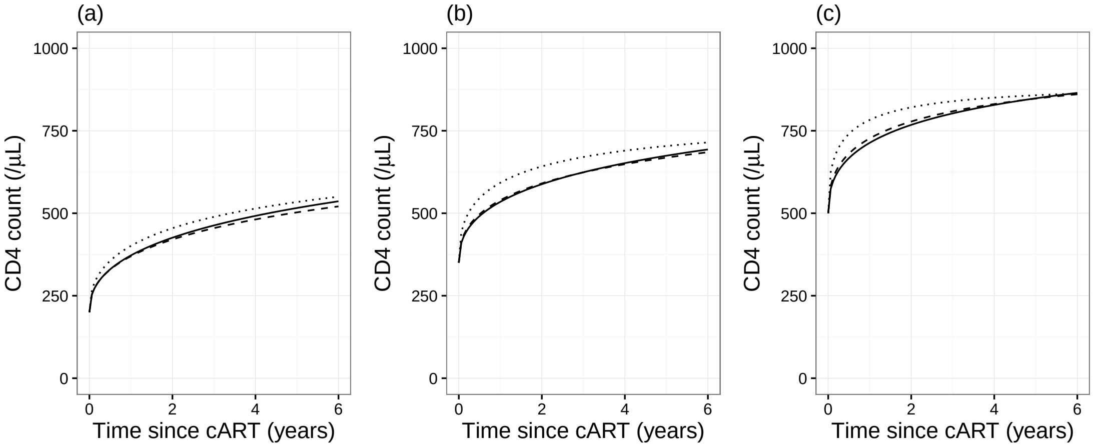

Figure 1.4. Plots of predicted median recovery in CD4 counts, based on Mod_{10}, for patients with a 'true' baseline value of 200 (a), 350 (b) or 500 (c) cells/μL. Predictions are shown for patients initiating treatment within 6 months of seroconversion (······), patients initiating treatment beyond 6 months but within 1 year (- - -) and for patients who started treatment beyond 1 year (——). For this plot, all patients are assumed to be men who have sex with men, aged 36 years, with negative test for hepatitis C virus, no prior AIDS diagnosis and starting on a non-nucleoside reverse-transcriptase inhibitor (NNRTI) regimen. Viral load prior to treatment is also fixed at the overall $\log_{10}$ median of 4.825. NOTE: this is presented as Figure 1 in the main paper.

tions. Of these patients, 2462 (32.6 %) had virological failure observed at some point in time, at a median of 0.90 years (IQR, 0.65–1.57 years).

When the set of models were fitted to the processed dataset with censoring of post-treatment CD4 counts at any occurrence of detectable viral load beyond 6 months after treatment initiation, the same pattern was observed of statistically significant improvements in model fit but with optimal BIC for the inclusion of only VL (Table 1.3). Furthermore, predictions generated from the fitted model including all patient and drug characteristics (i.e. Mod_{10}) are nearly identical to those for the equivalent model without censoring due to detectable VL (Figure 1.7). The parameter estimates for Mod_{10} fitted to the two versions of the dataset were correspondingly very similar, as can be seen in Table 1.2. There are some apparent differences between the parameter estimates relating to natural cubic spline functions, but plotting of the fitted functions reveals them to be similar within the range of values well represented in the data, for example the estimated functions linking baseline CD4 to recovery characteristics resulting from the model fitted to the censored dataset are plotted in Figure 1.8.

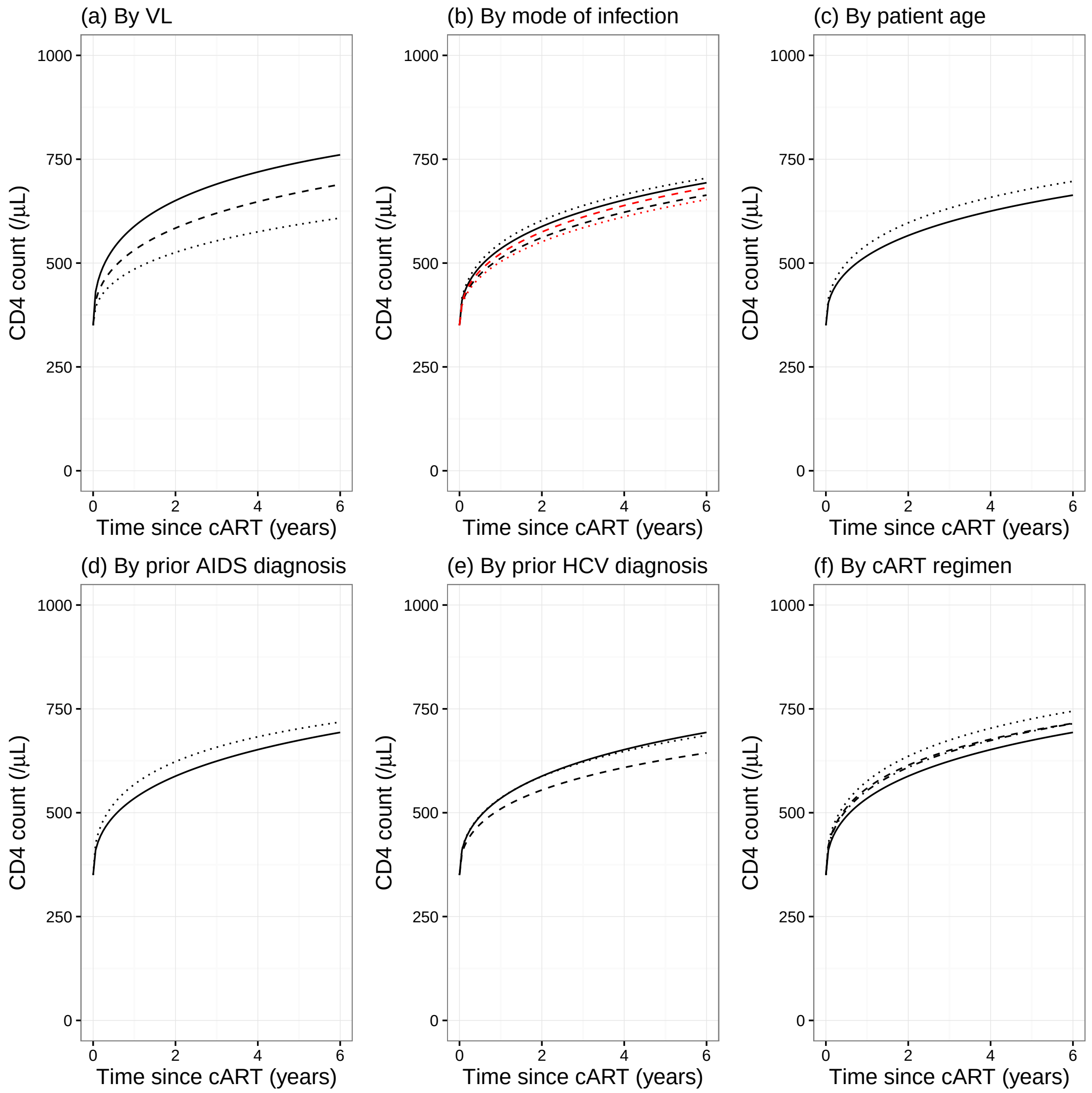

Figure 1.5. Plots of predicted median recovery in CD4 counts, based on Mod_{10}, for patients with a 'true' baseline value of 350 according to: (a) viral load (VL) prior to treatment initiation (······, $\log_{10}(\mathrm{VL}) = 2.7$; - - - , $\log_{10}(\mathrm{VL}) = 4.7$; ——, $\log_{10}(\mathrm{VL}) = 5.7$); (b) sex and infection groups (——, men who have sex with men; - - - , male heterosexual; ······, male injecting drug user; - - - , female heterosexual; ······, female injecting drug user); (c) patient age at treatment initiation (······, $age = 20$ years; ——, $age = 60$ years); (d) AIDS diagnosis prior to treatment (······, yes; ——, no); (e) hepatitis C virus (HCV) status (······, no test; - - - , +ve test; ——, -ve test); and (f) cART regimen (······, integrase strand transfer inhibitor; -·-·-, ritonavir-boosted protease inhibitor; - - - , other; ——, non-nucleoside reverse-transcriptase inhibitor (NNRTI)). All patients are assumed to be men who have sex with men, aged 36 years, with negative test for HCV, no prior AIDS diagnosis, baseline $\log_{10}$ (VL) of 4.825 and starting on a NNRTI regimen at more than 1 year since estimated date of seroconversion unless stated otherwise. NOTE: this is presented as Figure 2 in the main paper.

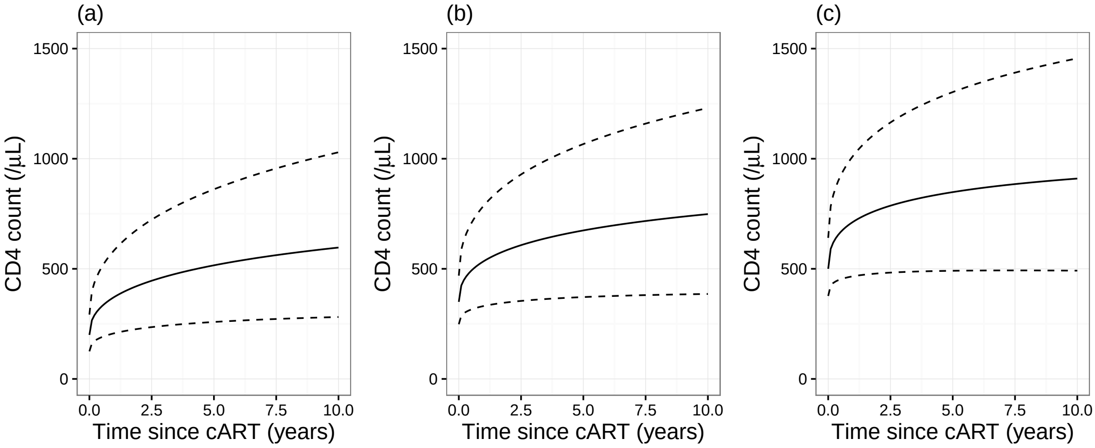

Figure 1.6. Plots of predicted median (——) and 5th and 95th centiles (- - -) for recovery in CD4 counts, based on Mod_{10}, for patients with a 'true' baseline value of 200 (a), 350 (b) or 500 (c) cells/μL. For this plot, all patients are assumed to be men who have sex with men, aged 36 years, with negative test for hepatitis C virus, no prior AIDS diagnosis and starting on a non-nucleoside reverse-transcriptase inhibitor (NNRTI) regimen beyond 1 year from seroconversion. Viral load prior to treatment is also fixed at the overall $\log_{10}$ median of 4.825. NOTE: this is presented as Figure 3 in the main paper.

Table 1.2. Parameter estimates for Mod_{10} as applied to the full CASCADE dataset, and to a processed dataset with censoring of CD4 counts at occurrence of detectable viral load (VL) beyond 6 months after treatment initiation.

Para.	Full data fit	Cens data fit	Para. (cont. 1)	Full data fit (cont. 1)	Cens data fit (cont. 1)	Para. (cont. 2)	Full data fit (cont. 2)	Cens data fit (cont. 2)
β_0	22.082 (21.955 to 22.209)	22.089 (21.962 to 22.216)	$Bt2_1$	-1.195 (-1.818 to -0.571)	-1.32 (-2.064 to -0.576)	FI_A	2.09 (-2.043 to 6.222)	5.05 (-0.135 to 10.235)
β_1	-1.456 (-1.524 to -1.388)	-1.462 (-1.531 to -1.393)	$Bt2_2$	0.025 (-0.019 to 0.069)	0.034 (-0.016 to 0.085)	FI_B	-0.381 (-0.89 to 0.128)	-0.597 (-1.106 to -0.089)
U_{00}	21.581 (20.638 to 22.567)	21.512 (20.569 to 22.499)	$Bt2_3$	0.009 (-0.061 to 0.078)	-0.002 (-0.069 to 0.064)	FH_A	1.026 (0.159 to 1.894)	2.044 (1.004 to 3.083)
ρ	-0.642 (-0.718 to -0.566)	-0.641 (-0.719 to -0.564)	$Bt2_4$	-0.003 (-0.145 to 0.14)	0.019 (-0.116 to 0.154)	FH_B	-0.166 (-0.273 to -0.059)	-0.256 (-0.374 to -0.138)
U_{11}	0.618 (0.482 to 0.791)	0.611 (0.474 to 0.787)	B_1	-1.375 (-1.797 to -0.954)	-1.139 (-1.614 to -0.664)	$ageA_1$	0.163 (0.033 to 0.293)	0.173 (0.029 to 0.317)
κ_{pre}	5.941 (5.619 to 6.281)	5.932 (5.602 to 6.281)	B_2	0.028 (-0.001 to 0.056)	0.016 (-0.016 to 0.049)	$ageA_2$	-0.039 (-0.085 to 0.007)	-0.048 (-0.099 to 0.004)
H_{pre}	0.321 (0.29 to 0.352)	0.327 (0.294 to 0.359)	B_3	-0.014 (-0.05 to 0.022)	-0.03 (-0.072 to 0.012)	$ageA_3$	0.095 (-0.035 to 0.226)	0.114 (-0.031 to 0.26)
σ	1.793 (1.759 to 1.827)	1.798 (1.762 to 1.834)	B_4	0.045 (-0.027 to 0.116)	0.082 (-0.002 to 0.165)	$ageA_4$	-0.08 (-0.207 to 0.046)	-0.09 (-0.231 to 0.051)
$At1_1$	23.399 (17.658 to 29.141)	24.838 (16.998 to 32.679)	P	9.929 (8.69 to 11.343)	11.083 (9.619 to 12.768)	$ageB_1$	-0.017 (-0.038 to 0.004)	-0.014 (-0.036 to 0.008)
$At1_2$	0.247 (-0.116 to 0.611)	0.153 (-0.346 to 0.652)	κ_{post}	4.608 (4.331 to 4.903)	3.879 (3.582 to 4.201)	$ageB_2$	0.002 (-0.004 to 0.009)	0.002 (-0.006 to 0.009)
$At1_3$	0.024 (-0.252 to 0.299)	0.119 (-0.225 to 0.462)	H_{post}	0.247 (0.229 to 0.265)	0.227 (0.205 to 0.25)	$ageB_3$	-0.004 (-0.023 to 0.015)	-0.002 (-0.022 to 0.018)
$At1_4$	-0.003 (-0.516 to 0.509)	-0.178 (-0.806 to 0.451)	D	0.423 (0.407 to 0.438)	0.426 (0.409 to 0.442)	$ageB_4$	0.002 (-0.016 to 0.02)	0 (-0.02 to 0.019)
$At2_1$	22.852 (16.221 to 29.483)	23.751 (15.427 to 32.076)	VA_1	0.292 (-0.727 to 1.31)	0.042 (-1.064 to 1.147)	$AIDS_A$	-2.052 (-3.296 to -0.808)	-2.959 (-4.205 to -1.713)
$At2_2$	0.394 (-0.051 to 0.84)	0.335 (-0.203 to 0.874)	VA_2	1.035 (-0.832 to 2.901)	1.556 (-0.453 to 3.565)	$AIDS_B$	0.423 (0.256 to 0.59)	0.547 (0.371 to 0.722)
$At2_3$	-0.062 (-0.557 to 0.434)	0.004 (-0.498 to 0.506)	VA_3	-5.334 (-12.038 to 1.369)	-7.599 (-14.83 to -0.369)	HCV_pre_A	-1.598 (-2.994 to -0.202)	-2.193 (-3.55 to -0.836)
$At2_4$	0.129 (-0.835 to 1.093)	-0.002 (-0.967 to 0.964)	VA_4	10.54 (-1.099 to 22.179)	14.643 (2.066 to 27.22)	HCV_pre_B	-0.004 (-0.209 to 0.201)	0.07 (-0.131 to 0.272)
A_1	25.192 (20.291 to 30.092)	21.304 (16.245 to 26.363)	VB_1	0.099 (-0.05 to 0.249)	0.121 (-0.029 to 0.272)	$no\_HCV\_test_A$	-1.079 (-1.657 to -0.502)	-0.722 (-1.349 to -0.094)
A_2	0.362 (0.042 to 0.682)	0.57 (0.23 to 0.909)	VB_2	-0.038 (-0.294 to 0.218)	-0.074 (-0.334 to 0.187)	$no\_HCV\_test_B$	0.114 (0.025 to 0.204)	0.086 (-0.007 to 0.179)
A_3	-0.038 (-0.371 to 0.295)	0.056 (-0.338 to 0.45)	VB_3	0.324 (-0.586 to 1.234)	0.476 (-0.457 to 1.41)	II_A	-0.727 (-1.72 to 0.266)	-0.864 (-1.91 to 0.182)
A_4	0.047 (-0.593 to 0.688)	-0.198 (-0.962 to 0.565)	VB_4	-0.353 (-1.946 to 1.24)	-0.554 (-2.204 to 1.095)	II_B	0.309 (0.152 to 0.465)	0.327 (0.167 to 0.486)
$Bt1_1$	-1.358 (-1.969 to -0.746)	-1.699 (-2.541 to -0.858)	MI_A	-1.043 (-3.248 to 1.162)	-0.139 (-2.568 to 2.289)	PrI_A	-0.714 (-1.339 to -0.088)	-0.651 (-1.329 to 0.027)
$Bt1_2$	0.061 (0.019 to 0.103)	0.085 (0.028 to 0.143)	MI_B	0.188 (-0.132 to 0.508)	0.082 (-0.258 to 0.422)	PrI_B	0.182 (0.102 to 0.263)	0.18 (0.095 to 0.266)
$Bt1_3$	0.026 (-0.027 to 0.079)	-0.005 (-0.067 to 0.058)	MH_A	1.124 (0.112 to 2.136)	1.193 (0.09 to 2.296)	$other_A$	-1.375 (-2.526 to -0.224)	-1.117 (-2.358 to 0.124)
$Bt1_4$	-0.044 (-0.151 to 0.063)	0.012 (-0.112 to 0.135)	MH_B	-0.245 (-0.37 to -0.12)	-0.256 (-0.393 to -0.119)	$other_B$	0.288 (0.115 to 0.461)	0.244 (0.066 to 0.423)

95% CIs are given in parentheses. Parameters (Para.) up to H_{post} are as described in Table 2 of Stirrup *et al.*[1]. D is the shape parameter for the Janoshek–Sager curve. VA_i parameters relate to the natural cubic spline (NCS) function for the effect of pre-treatment viral load on long-term maximum recovery. MH_A, MI_A, FH_A and FI_A denote the effect of being a male heterosexual, male injecting drug user (IDU), female heterosexual or female IDU, respectively. $ageA_i$ are NCS function parameters for patient age at treatment initiation. Estimated effects are also shown for occurrence of an AIDS diagnosis prior to treatment ($AIDS_A$), a positive hepatitis C virus test (HCV_pre_A) or lack of test before treatment ($no\_HCV\_test_A$), and for cART initiation on an integrase strand transfer inhibitor regimen ($INSTI_A$), ritonavir-boosted protease inhibitor regimen (PI_A) or 'other' regimen ($other_A$). Equivalent 'B' parameters denote the corresponding estimated effect of the speed of recovery following treatment initiation.

Table 1.3. Summary of fitted combined models for CD4 cell counts before and after the initiation of combination antiretroviral therapy (cART) in patients from the CASCADE cohort, with censoring of post-treatment CD4 counts at the observation of detectable viral load beyond 6 months after treatment initiation. All models shown are nested within that described in the row below.

Model	Predictors	Curve	n_{pars}	ℓ	AIC	BIC	$2\Delta\ell$
Mod_1	Linear-u	Asym.	15	-205 209	410 448	410 588	NA
Mod_2	Linear-u	JS	16	-204 132	408 296	408 445	2 154
Mod_3	As above + trt-time grp	JS	24	-203 806	407 660	407 884	652
Mod_4	As above + baseline VL	JS	32	-203 486	407 036	407 335*	640
Mod_5	As above + gender/inf grp	JS	40	-203 468	407 016	407 389	36
Mod_6	As above + age	JS	48	-203 453	407 002	407 450	30
Mod_7	As above + AIDS Dx	JS	50	-203 437	406 974	407 441	32
Mod_8	As above + HCV Dx	JS	54	-203 426	406 960	407 464	22
Mod_9	As above + trt class	JS	60	-203 400	406 920	407 480	52
Mod_{10}	As above + NCS-u	JS	72	-203 369	406 882*	407 554	62

The 'Predictors' field lists variables included in the functions to determine both long-term maximum (ϕ_1) and speed of recovery (ϕ_2), and 'Curve' gives shape of expected recovery following cART. 'trt-time grp' denotes stratification of functions for long-term maximum and speed of recovery in terms of baseline CD4 at treatment initiation. *Lowest value of AIC/BIC for set of models. '$2\Delta\ell$' denotes differences in 2×log-likelihood in comparison to model described in the row above in each case. AIC, Akaike information criterion; AIDS, acquired immune deficiency syndrome; Asym., asymptotic; BIC, Bayesian information criterion; Dx, diagnosis prior to cART; HCV, hepatitis C virus; grp, group; inf, mode of infection; JS, Janoshek–Sager; ℓ, log-likelihood of model fit; NA, not applicable; NCS, natural cubic spline; n_{pars}, number of parameters in model; trt, treatment; VL, viral load.

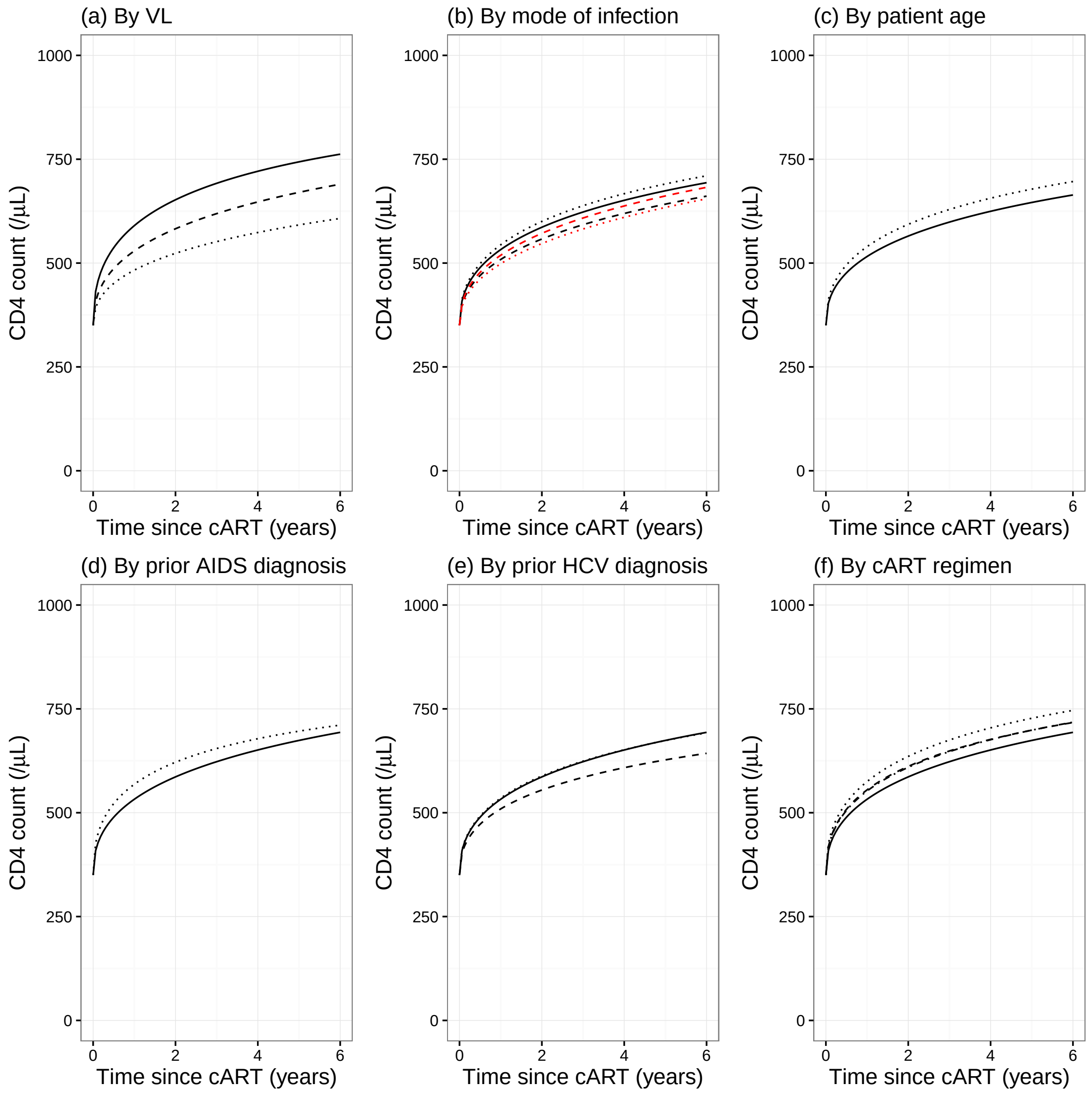

Figure 1.7. Plots of predicted median recovery in CD4 counts, based on Mod_{10} fitted to the dataset with censoring at detectable viral load (VL) after 6 months of treatment, for patients with a 'true' baseline value of 350 according to: (a) VL prior to treatment initiation (······, $\log_{10}(\text{VL}) = 2.7$; - - - , $\log_{10}(\text{VL}) = 4.7$; ——, $\log_{10}(\text{VL}) = 5.7$); (b) sex and infection groups (——, men who have sex with men; - - - , male heterosexual; ······, male injecting drug user; - - - , female heterosexual; ······, female injecting drug user); (c) patient age at treatment initiation (······, $age = 20$ years; ——, $age = 60$ years); (d) AIDS diagnosis prior to treatment (······, yes; ——, no); (e) hepatitis C virus (HCV) status (······, no test; - - - , +ve test; ——, -ve test); and (f) cART regimen (······, integrase strand transfer inhibitor; -·-·-, ritonavir-boosted protease inhibitor; - - - , other; ——, non-nucleoside reverse-transcriptase inhibitor (NNRTI)). All patients are assumed to be men who have sex with men, aged 36 years, with negative test for HCV, no prior AIDS diagnosis, baseline $\log_{10}$ (VL) of 4.825 and starting on a NNRTI regimen at more than 1 year since estimated date of seroconversion unless stated otherwise.

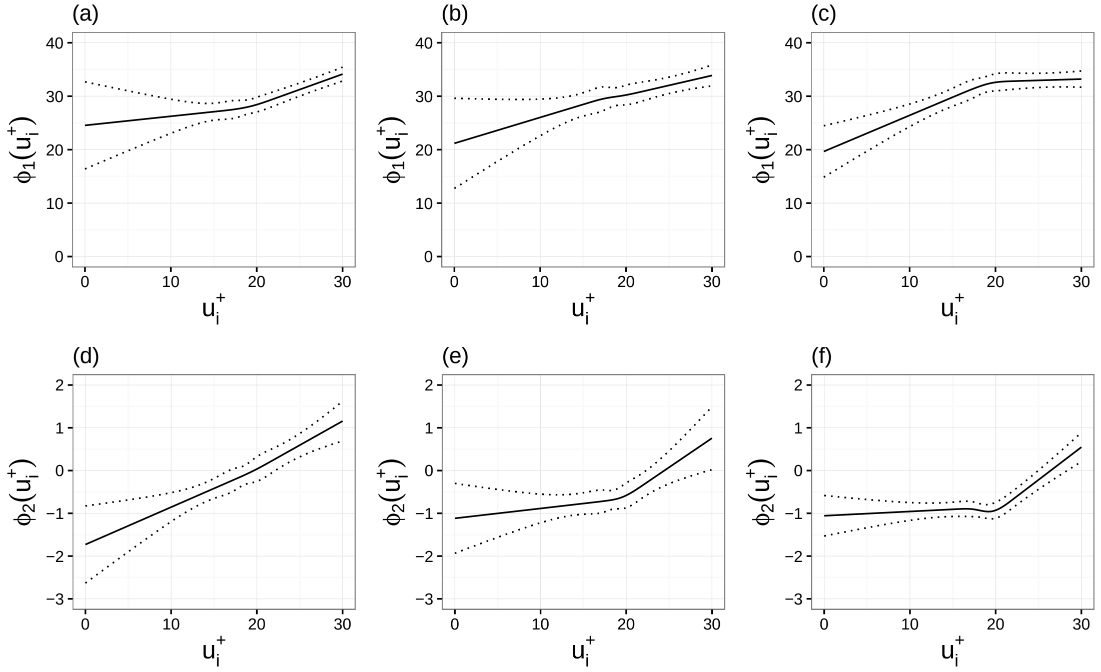

Figure 1.8. Plots of $\phi_1\left(u_i^+\right)$ (a–c, relating to long-term maximum) and $\phi_2\left(u_i^+\right)$ (d–f, relating to speed of response) for Mod_{10} fitted to the dataset with censoring at detectable viral load (VL) after 6 months of treatment. Graphs on the left of each row (a,d) show the fitted functions for patients initiating treatment within 6 months of seroconversion, those in the centre (b,e) show the functions for patients initiating treatment beyond 6 months but within 1 year and those on the right (c,f) show the functions for patients who started treatment beyond 1 year. Pointwise 95 % confidence intervals for the functions are shown (······).

2 Modelling uncertainty in seroconversion date

In Section 1 of this Appendix, we describe models based on the assumption that the estimated date of seroconversion in each patient is correct. However, the uncertainty in exact date of seroconversion for those patients with a 'mid-point' estimate, set at the halfway point between last negative and first positive HIV tests, raises questions regarding the interpretation of any fitted models, particularly when trying to determine whether treatment initiation close to the date of seroconversion might lead to substantial improvements in CD4 recovery.

There has been development of statistical methodology to address the problem of uncertainty in seroconversion dates, both in order to provide more accurate estimation of infection time in 'seroconverters' and to allow modelling of the delay to diagnosis in 'seroprevalent' patients. Early work on this issue was motivated by the need to estimate the survival function for the progression from seroconversion to AIDS (before the availability of effective treatment)[6;7], while more recent research has focused on the need for accurate estimation of infection dates for monitoring of the incidence of new HIV cases in different countries and communities[8;9]. One approach to dealing with the problem is to define a time-to-event regression model for the interval between seroconversion and first observation of a patient, with biomarkers at presentation used as predictive variables; for example, Muñoz *et al.*[6] used a truncated Weibull regression model for the time elapsed since seroconversion among 'seroconverters' with CD4 % as a predictive variable, and then used the results to impute dates for seroprevalent patients. Geskus[7] proposed a non-parametric approach in which the estimated distribution of the timing of seroconversion, conditional on observed CD4 counts, is empirically derived based on data from patients with well estimated date of seroconversion (including patients with an interval of up to 1 year between negative and positive tests).

Taffé *et al.*[8] developed a joint model incorporating time from seroconversion to diagnosis, differences between serial CD4 count measurements following diagnosis and drop-out from the analysis due to either ART or death. Estimation of the parameters for this model requires integration over correlated subject-specific random effect terms for the intercept and slope of CD4 trajectory as well as an independent measurement error term (the latter resulting from the fact that the model for CD4 counts is defined in terms of differences from the first observation) in order to obtain the marginal log-likelihood. In this model, the time from seroconversion to diagnosis is treated as a time-to-event outcome variable, the model for which is defined conditional on the random effect terms. Conditional imputation of the date of seroconversion for any given patient therefore requires calculations based on the empirical Bayes estimates of their random effects.

We approach this problem through the further extension of methodology proposed by Sommen *et al.*[9], who developed longitudinal models for virological markers of recent infection in which the time elapsed from seroconversion to diagnosis for each patient is itself treated as a latent variable. This has the advantage that the models for the biomarkers under investigation can be defined in terms of the true time since seroconversion, with the marginal log-likelihood obtained by integration over the range of possible seroconversion dates for any given patients as well as any subject-specific random effects. A similar approach was independently described by Drylewicz *et al.*[10], who developed dynamic models for pre-treatment CD4 cell counts and viral load measurements in HIV patients with integration of the likelihood function over possible infection dates. The markers under investigation by Sommen *et al.* were antibodies to the immunodominant epitope of gp41 (IDE) and a mixture of five V3 peptides; their model for each comprised an asymptotic increase from zero and independent Brownian motion and measurement error terms, and a uniform prior distribution was assumed for the occurrence of seroconversion between last negative and first positive HIV tests (or over an interval of 70 days prior to signs of symptomatic primary infection or 30 days prior to incomplete Western blot).

In this Section, we develop a model for pre-treatment CD4 counts and viral load measurements and for the recovery in CD4 counts following initiation of cART conditional on the true date of seroconversion for each patient. We follow the principle proposed by Sommen *et al.*[9] and Drylewicz *et al.*[10] of obtaining the marginal log-likelihood by integration over a prior distribution of the true date for each patient informed by the interval between negative and positive tests, although beyond this the model structure that we develop differs from their work. Our work is novel in that we also model response to cART in terms of the true time elapsed from seroconversion to initiation of treatment.

2.1 Exact seroconversion date as a latent variable

If a distribution can be assigned for the true seroconversion date t' for each patient, with probability density function $f_{t'}$, then the marginal likelihood for an extension of the combined model for pre- and post-treatment data as described in Stirrup *et al.*[1] can be expressed as follows:

$$f\left(\mathbf{y}_{pre},\mathbf{y}_{post}\right) = \int_{t'_{min}}^{t'_{max}} \int_{-\infty}^{\infty} f_{pre}\left(\mathbf{y}_{pre}|T'=t'\right) f_{post}\left(\mathbf{y}_{post}|u,T'=t'\right) f_u\left(u|\mathbf{y}_{pre},T'=t'\right) f_{t'}\left(t'\right) du\, dt'.$$

This integral is of a form similar to that used by Sommen *et al.*[9].

The Laplace approximation to the marginal likelihood is optimally accurate for latent variables integrated out over a normal scale[11], and so for maximum likelihood estimation we instead express this integral as:

$$f\left(\mathbf{y}_{pre}, \mathbf{y}_{post}\right) = \int_{-\infty}^{\infty}\int_{-\infty}^{\infty} f_{pre}\left(\mathbf{y}_{pre}|T' = F_{t'}^{-1}\left(\Phi\left(a\right)\right)\right) f_{post}\left(\mathbf{y}_{post}|u, T' = F_{t'}^{-1}\left(\Phi\left(a\right)\right)\right)$$
$$f_{u}\left(u|\mathbf{y}_{pre}, T' = F_{t'}^{-1}\left(\Phi\left(a\right)\right)\right) f_{\phi}\left(a\right) du\, da,$$

where f_{ϕ} is the probability density function for a standard normal distribution and $F_{t'}^{-1}$ is the inverse of the cumulative distribution function corresponding to $f_{t'}$.

The expression used by ADMB for the gradient of the Laplace approximation to an integral involves third order partial derivatives of the integrand with respect to the latent variable terms, which means that the response to treatment cannot be modelled according to arbitrary divisions of the time from 'true date of seroconversion' to treatment initiation; for such models the integrand would not be differentiable with respect to t' across its range of potential values. For this extension to the model, we hypothesise that the response in CD4 count to cART follows distinct relationships with the baseline value 'u^{+}' according to whether treatment is initiated very close to the date of seroconversion or after a long period of time has elapsed. As described in Stirrup *et al.*[1], the response to treatment is modelled as being dependent on the baseline CD4 value through functions that determine the expected long-term maximum and speed of recovery, with separate functions defined for 'early' and 'late' treatment initiation (denoted $\phi_{1:early}(u^{+})$, $\phi_{2:early}(u^{+})$, $\phi_{1:late}(u^{+})$ and $\phi_{2:late}(u^{+})$). However, unlike in Stirrup *et al.*[1], we incorporate a smooth transition from the 'early' to the 'late' functions according to the exact value of t', by weighting their respective contributions towards the expected long-term maximum $\phi_{1:i}$ or speed of response $\phi_{2:i}$ for any given patient according to the functions:

$$weight_{early:i} = 2 - 2/\left(1 + \exp\left(-S * t_{trt:i}\right)\right)$$
$$weight_{late:i} = 2/\left(1 + \exp\left(-S * t_{trt:i}\right)\right) - 1,$$

here S is a parameter to be estimated that determines the balance between 'early' and 'late' treatment response characteristics according to the time elapsed between true date of seroconversion and initiation of cART '$t_{trt:i}$'. These two weighting function sum to 1 for any value of $t_{trt:i}$, for $t_{trt:i} = 0$ the functions return 1 and 0, and $weight_{early:i} \rightarrow 0$ and $weight_{late:i} \rightarrow 1$ as $t_{trt:i}$ increases.

2.2 Incorporating viral load into the model

Following our aim of investigating the separate contributions of baseline CD4 count, time from seroconversion to treatment initiation and baseline viral load in predicting the characteristics of CD4 recovery on cART, we also extend the model to include pre-treatment viral load as an outcome variable. This development is necessary in order to allow information from viral load observations to contribute to the posterior distribution of the true date of seroconversion for each patient, and it also means that patients for whom no viral load observations were obtained close to the start of treatment can be included in the analysis. Viral load is analysed on the $\log_{10}$ scale, and we make use of the non-linear model for the mean in terms of time from seroconversion as reported by Pantazis *et al.*[5]:

$$g_{VL}(t_{VL}) = \beta_{0VL} + \beta_{1VL} t_{VL} + \beta_{2VL} \exp\left(-\beta_{3VL} t_{VL}\right), \tag{1}$$

where t_{VL} is the time of VL observation from date of seroconversion and β_{0VL}–β_{3VL} are parameters to be estimated.

However, a patient-specific random effect is only included for the intercept and not for the long-term slope, as we were unable to successfully fit models that also included the latter term (the program crashed or convergence failed). The patient-specific random intercept is modelled as following a joint multivariate normal distribution with the random-intercept and -slope terms of the pre-treatment CD4 part of the model, and there is also an examination-specific independent normal error term for the pre-treatment viral load:

$$
\begin{aligned}
\mathbf{v}_i &= \mathbf{g}_{VL}(\mathbf{t}_{VL:i}) + \mathbf{1}\mathrm{b}_{VL:i} + \mathbf{e}_{VL:i} \\
\mathbf{y}_{pre:i} &= \mathbf{X}_i \boldsymbol{\beta} + \mathbf{Z}_i \mathbf{b}_i + \mathbf{W}_{pre:i} + \mathbf{e}_{pre:i} \\
\begin{pmatrix} \mathrm{b}_{VL:i} \\ \mathbf{b}_i \end{pmatrix} &\sim MVN\left(\mathbf{0}, \begin{pmatrix} \psi_{VL} & \mathrm{Cov}(\mathrm{b}_{VL:i}, \mathbf{b}_i) \\ \mathrm{Cov}(\mathbf{b}_i, \mathrm{b}_{VL:i}) & \boldsymbol{\Psi} \end{pmatrix}\right) \\
\mathbf{e}_{VL:i} &\sim MVN(\mathbf{0}, \sigma^2_{VL} \mathbf{I}_{n_{VL:i}}) \\
\mathbf{W}_{pre:i} &\sim MVN(\mathbf{0}, \boldsymbol{\Sigma}_{pre:i}) \\
\mathbf{e}_{pre:i} &\sim MVN(\mathbf{0}, \sigma^2 \mathbf{I}_{n_{pre:i}}).
\end{aligned}
$$

Here, $\mathbf{v}_i$ is the vector of $n_{VL:i}$ pre-treatment viral load observations for the ith patient at times $\mathbf{t}_{VL:i}$, $\mathbf{g}_{VL}$ is a vectorised version of the function in (1), $\mathbf{1}$ is a vector of '1's of length $n_{VL:i}$, $\mathrm{b}_{VL:i}$ is the subject-specific random intercept for viral load with variance ψ_{VL}, $\mathbf{e}_{VL:i}$ is a vector of examination-specific residuals for viral load with variance σ^2_{VL}, and other terms are as defined in Stirrup *et al.*[1]. The time values in this model are defined with respect to the true date of seroconversion for each patient

through conditioning on the corresponding latent variable term.

A further complication is that the viral load measurements recorded in the CASCADE dataset are truncated at lower and upper limits of detection, with these limits depending on the equipment used at each examination and ranging from 1–500 copies/mL for the lower limit and 50 000–10^8 copies/mL for the upper limit. Following Thiébaut *et al.*[12;13], we account for this issue by making use of the fact that the likelihood contribution for such an observation below a lower limit of detection, conditional on the subject-specific random intercept, is independent of other observations and can be expressed using the cumulative normal distribution function (Φ)[14] and the lower limit of detection in that case (lim_{ij}^{L}):

$$L(v_{ij}|\mathrm{b}_{VL:i}) = \Phi\left(\left(lim_{ij}^{L} - \left(g_{VL}\left(t_{VL:ij}\right) + \mathrm{b}_{VL:i}\right)\right)/\sigma_{VL}\right),$$

while the likelihood contribution for observations above the upper limit of detection can be expressed using the upper limit (lim_{ij}^{U}) in that case:

$$L(v_{ij}|\mathrm{b}_{VL:i}) = 1 - \Phi\left(\left(lim_{ij}^{U} - \left(g_{VL}\left(t_{VL:ij}\right) + \mathrm{b}_{VL:i}\right)\right)/\sigma_{VL}\right).$$

This has the consequence that approximation of the marginal log-likelihood requires integration over the viral load random intercept term for each patient. If there were no lower limits of detection, then it would be possible to form a joint multivariate normal distribution (with associated closed form probability density function) for both the CD4 count and viral load observations in the pre-treatment part of the model. However, we may still express the probability density function for the pre-treatment CD4 count observations in closed form conditional on the viral load random intercept term in each patient, making use of standard expressions for conditional normal distributions. If we express the joint distribution for the viral load and CD4 random effects as follows:

$$\begin{pmatrix} \mathrm{b}_{VL:i} \\ \mathbf{b}_i \end{pmatrix} \sim MVN\left(\mathbf{0}, \begin{pmatrix} \psi_{VL} & \boldsymbol{\psi}_{12} \\ \boldsymbol{\psi}_{21} & \boldsymbol{\Psi} \end{pmatrix}\right),$$

then the conditional model for the pre-treatment CD4 counts can be expressed as:

$$\begin{aligned}
\mathbf{y}_{pre:i}|\mathrm{b}_{VL:i} &= \mathbf{X}_i\boldsymbol{\beta} + \mathbf{Z}_i\mathbf{b}_i + \mathbf{W}_{pre:i} + \mathbf{e}_{pre:i} \\
\mathbf{b}_i|\mathrm{b}_{VL:i} &\sim MVN\left(\frac{\boldsymbol{\psi}_{21}\mathrm{b}_{VL:i}}{\psi_{VL}}, \boldsymbol{\Psi} - \frac{\boldsymbol{\psi}_{21}\boldsymbol{\psi}_{12}}{\psi_{VL}}\right) \\
\mathbf{W}_{pre:i} &\sim MVN(\mathbf{0}, \boldsymbol{\Sigma}_{pre:i}) \\
\mathbf{e}_{pre:i} &\sim MVN(\mathbf{0}, \sigma^2\mathbf{I}_{n_{pre:i}}).
\end{aligned}$$

We also allow the post-treatment recovery in CD4 cell counts to be dependent on the realisation of b_{VL}, and the marginal log-likelihood for the complete model can therefore be expressed as:

$$\begin{aligned}
f\left(\mathbf{y}_{pre},\mathbf{v},\mathbf{y}_{post}\right) = \int_{-\infty}^{\infty}\int_{-\infty}^{\infty}\int_{-\infty}^{\infty} & f_{pre}\left(\mathbf{y}_{pre}|T' = F_{t'}^{-1}\left(\Phi\left(a\right)\right),\mathrm{b}_{VL}\right) \\
& f_{post}\left(\mathbf{y}_{post}|u, T' = F_{t'}^{-1}\left(\Phi\left(a\right)\right),\mathrm{b}_{VL}\right) \\
& f_{VL}\left(\mathbf{v}|T' = F_{t'}^{-1}\left(\Phi\left(a\right)\right),\mathrm{b}_{VL}\right) \\
& f_{u}\left(u|\mathbf{y}_{pre}, T' = F_{t'}^{-1}\left(\Phi\left(a\right)\right),\mathrm{b}_{VL}\right) \\
& f_{\mathrm{b}_{VL}}\left(\mathrm{b}_{VL}\right) f_{\phi}\left(a\right) du\ da\ d\mathrm{b}_{VL}.
\end{aligned} \tag{2}$$

A directed acyclic graph to demonstrate the structure of this model is presented in Figure 2.1. As for Figure 3 in Stirrup *et al.*[1], links in this graph represent dependencies in the defined probability model rather than direct causal effects.

In the models that we present, the patient-specific random intercept for VL is included as a linear predictor for the long-term maximum (ϕ_1) and speed of recovery (ϕ_2) of post-treatment CD4 counts. As described in Section 2.1, parameters are fitted corresponding to early and late treatment initiation, with the weighting of the two for each patient dependent on the exact time elapsed from seroconversion to treatment initiation (which is itself defined in terms of a latent variable for those patients in whom date of seroconversion is known to fall within an interval between positive and negative tests). The use of the patient-specific VL intercept as a predictor of CD4 recovery (rather than the absolute VL level) means that the parameter estimates can be interpreted in terms of the patient's VL relative to the distribution across the population at any given point in time following seroconversion.

In approximating the marginal likelihood for this model, using the integral as shown in (2), greatest weight is placed on the values for the true date of seroconversion that maximise the joint penalised likelihood function that forms the integrand; this includes the probability density function for the pre-treatment viral load observations, as well as the prior distribution for the true date of seroconversion and the probability density functions corresponding to the pre- and post-treatment and baseline CD4 counts. All of these aspects of the model as a whole can influence the posterior distribution of the true date of seroconversion for each patient, and so the component of the model relating to pre-treatment viral load measurements could affect estimates of how the post-treatment recovery in CD4 counts varies according to other factors such as time elapsed from seroconversion to treatment initiation.

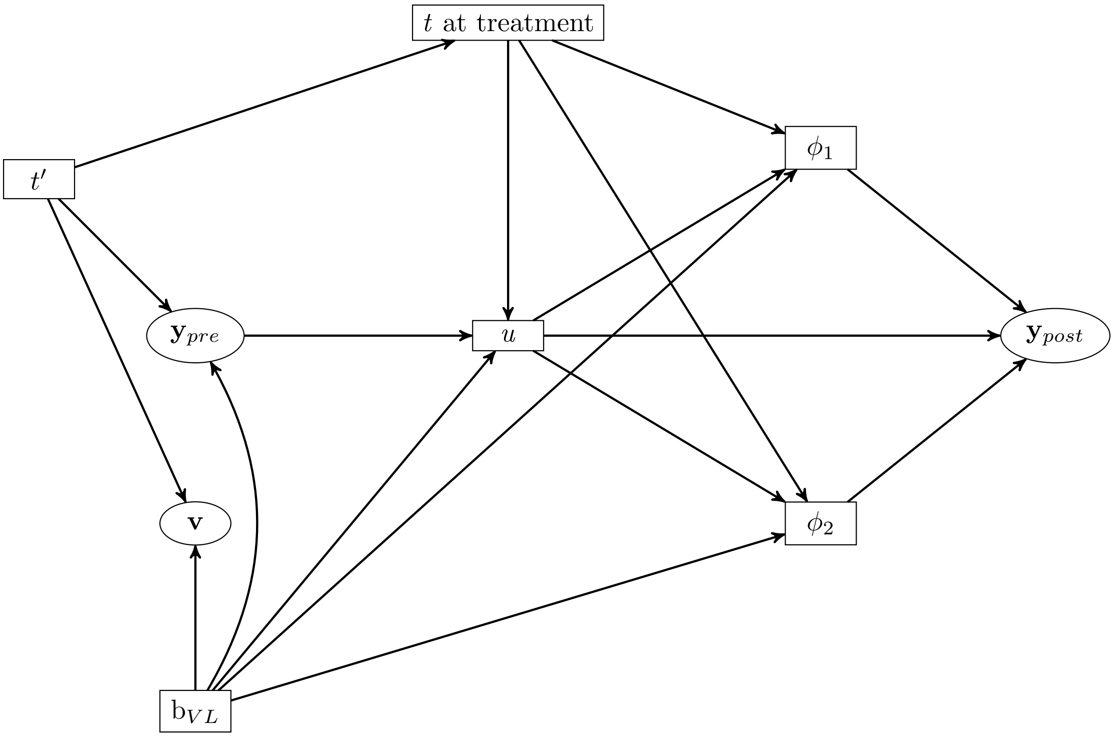

Figure 2.1. Directed acyclic graph depicting the proposed model structure for each patient, accounting for uncertainty in true date of seroconversion (t') and incorporating a probability model for pre-treatment viral load (VL). The distributions for both viral load observations ($\mathbf{v}$) and pre-treatment CD4 counts ($\mathbf{y}_{pre}$) are conditioned on the value of a random intercept variable for VL (b_{VL}), which also influences the long-term maximum (ϕ_1) and speed of recovery (ϕ_2) of post-treatment CD4 counts ($\mathbf{y}_{post}$). The distribution of the 'true' CD4 count at treatment initiation (u) is conditional on the pre-treatment observations and the timing of treatment, and the value of b_{VL} also affects the joint distribution of $\mathbf{y}_{pre}$ and u. Observed variables are shown within ellipses, whilst unobserved latent variables are shown within rectangles.

2.3 Prior distribution for true date of seroconversion

We define the 'prior distribution' of true seroconversion dates as that expected before consideration of any CD4 count or viral load data. For those patients in whom seroconversion date has been estimated as the midpoint between the last negative and first positive HIV tests, an obvious choice for the prior distribution is a uniform distribution over the interval between tests. However, we found that models using a uniform distribution would not converge, and so instead use a beta distribution scaled to match the duration of the interval between tests with alpha and beta parameters both fixed at 6. The use of this distribution makes the assumption that seroconversion is most likely to have occurred close to the midpoint between negative and positive tests. This assumption may not be completely justified, but the model nonetheless represents an improvement over the common assumption that seroconversion date is fixed at the midpoint. Plots illustrating these different assumptions for the prior distribution of true seroconversion dates are shown in Figure 2.2. For those

patients in whom the date of seroconversion illness or lab evidence of seroconversion (real-time PCR positivity or incomplete Western blot) is recorded, the date of seroconversion was considered to be fixed and known.

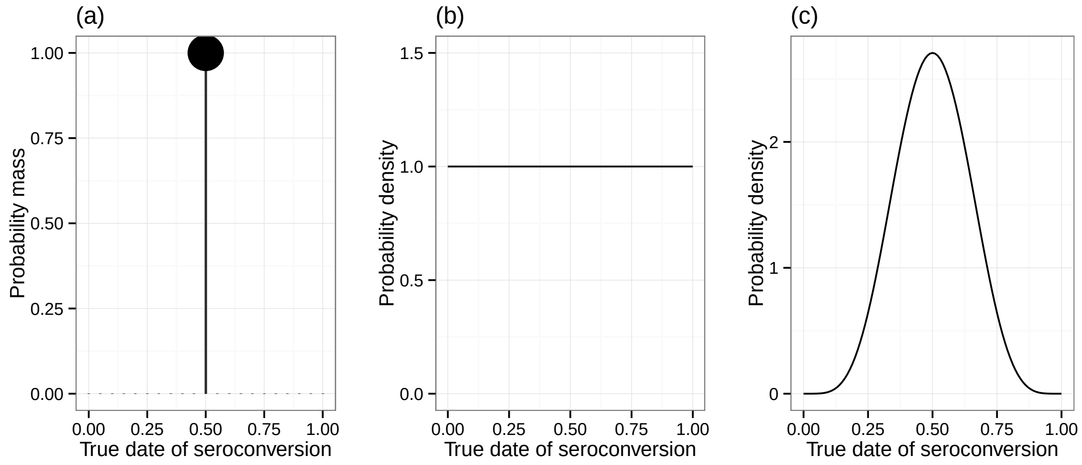

Figure 2.2. Plots illustrating different assumptions for the prior distribution of true seroconversion dates in a patient with their date of seroconversion estimated according to the interval between last negative and first positive HIV tests: (a) mid-point assumption, (b) uniform distribution and (c) beta distribution with alpha=beta=6. An interval between tests of 1 year is shown here, with the x-axis representing the true date of seroconversion within this period (i.e. 0 denotes date of last negative test).

For computational reasons, we also shift the distribution of possible seroconversion dates back in time by 1 day for all patients. This is required because ADMB-generated programs return 'not a number' when asked to return '0^c' for any value of 'c', which causes problems when calculating the covariance terms relating to fractional Brownian motion processes for each patient (involving 't^{2H}' terms). When time is fixed the issue can be avoided by defining a new function that checks that the base is not zero before attempting to calculate a power term, but this is not possible when time is allowed to vary within patients.

2.4 Dataset and estimation

The CASCADE dataset includes patients with a gap between last negative and first positive test of up to 3 years. For this analysis, we apply the same inclusion criteria as specified in the main paper (as applied in Section 1), except that patients are not excluded if they lack any VL observations within 6 months before treatment initiation; this is because VL is being included as a modelled outcome variable and so can be effectively imputed for patients in whom no measurements are available. This leads to a higher number of patients for potential inclusion in the analysis ($n = 8471$). However, to ensure the coherence of the proposed model we also exclude patients in whom the date of seroconversion was estimated according to the mid-point method

but who initiated cART before their first positive HIV test is recorded in the database ($n = 95$), leading to a study population of 8376 patients. Similarly, we remove from the analysis any CD4 counts that are recorded before the first positive HIV test. This results in a dataset of 41 456 pre-treatment CD4 counts, 66 932 post-treatment CD4 counts and 38 295 pre-treatment VL measurements.

Of the patients included in this analysis, 6521 (77.9 %) had an estimated date of seroconversion based on the mid-point between last negative and first positive HIV tests, for 1578 (18.8 %) it was based on laboratory evidence of seroconversion and in 277 (3.3 %) it was based on the reported date of seroconversion illness. For those patients in whom a 'mid-point' estimate of seroconversion date was used, the median interval between tests was 309 days and the IQR was 161–549 days.

As in Stirrup *et al.*[1] and Section 1 of this Appendix, maximum likelihood estimation was carried out using the random effects mode of the ADMB software, run on the UCL Legion High Performance Computing Facility. The Laplace approximation to the marginal log-likelihood was used for all models fitted in this Section. The correlations between random effect terms were parameterised using the Cholesky factor to ensure that the covariance matrix for the joint distribution of $\mathbf{b}_i$ and $\mathrm{b}_{VL:i}$ would remain positive-definite during optimisation, because of this indirect parameterisation confidence intervals are not presented for the estimated correlations.

2.5 Results

The models fitted to the full CASCADE dataset (without censoring related to post-treatment VL) are summarised in Table 2.1. The base-model (Mod_1') for this section of the analysis includes baseline CD4 and the patient-specific VL random intercept as linear predictors, with 'early' and 'late' parameters weighted as described in Section 2.1, for long-term maximum and speed of recovery of post-treatment CD4 counts; recovery follows a Janoshek–Sager curve with constant D parameter (as defined in Subsection 1.3). Although it should be noted that use of the Laplace approximation for the marginal log-likelihood means that assessment of AIC and BIC statistics requires caution, the addition of further patient and drug regimen characteristics in Mod_2'–Mod_6' led to only moderate improvements in model fit, as was found in Section 1 of this Appendix. Convergence of maximum likelihood estimates of model parameters was not achieved when natural cubic spline functions were used to provide more flexible link functions between baseline CD4 and VL and the characteristics of post-treatment recovery in CD4, and so all of the results presented in this Section follow from models in which these variables are treated as linear predictors. In the final model listed in Table 2.1, Mod_7', the D parameter relating to the Janoshek–Sager curve was also allowed to vary according to the time elapsed from seroconversion to

treatment initiation, with weighting of 'early' and 'late' parameters on the natural-log scale used for optimisation. We interpret this model in order to provide a comparison with the 'fixed estimate of seroconversion' analysis in Section 1.

Table 2.1. Summary of fitted combined models for CD4 cell counts before and after the initiation of combination antiretroviral therapy (cART) in patients from the CASCADE cohort, incorporating pre-treatment viral load measurements and uncertainty in the timing of seroconversion. All models shown are nested within that described in the row below.

Model	Predictors	n_{pars}	ℓ †	AIC†	BIC†	$2\Delta\ell$†
Mod_1^l	Linear-u + RNA w/ trt-time grp	33	−304 746	609 558	609 885*	NA
Mod_2^l	As above + gender/inf grp	41	−304 727	609 536	609 942	38
Mod_3^l	As above + age	49	−304 716	609 530	610 015	22
Mod_4^l	As above + AIDS Dx	51	−304 711	609 524	610 029	10
Mod_5^l	As above + HCV Dx	55	−304 701	609 512	610 056	20
Mod_6^l	As above + trt class	61	−304 686	609 494	610 098	30
Mod_7^l	As above + D by trt-time	62	−304 669	609 462*	610 076	34

The 'Predictors' field lists variables included in the functions to determine both long-term maximum (ϕ_1) and speed of recovery (ϕ_2). 'trt-time' denotes weighting of functions for long-term maximum and speed of recovery, in terms of baseline CD4 and VL at treatment initiation, according to time elapsed from seroconversion to treatment; the D parameter is treated as a function of time to treatment in Mod_7^l. *Lowest value of AIC/BIC for set of models. †All calculations using the log-likelihood for each model are based on the Laplace approximation. '$2\Delta\ell$' denotes differences in 2×log-likelihood in comparison to model described in the row above in each case. AIC, Akaike information criterion; AIDS, acquired immune deficiency syndrome; BIC, Bayesian information criterion; Dx, diagnosis prior to cART; HCV, hepatitis C virus; grp, group; inf, mode of infection; ℓ, log-likelihood of model fit; NA, not applicable; n_{pars}, number of parameters in model; trt, treatment.

In the models in this Section, the characteristics of CD4 recovery on cART follow a smooth function of the time elapsed from seroconversion to initiation of treatment with a transition from an 'early treatment' to a 'late treatment' response conditional on baseline CD4 and VL random intercept. The transition as estimated for Mod_7^l is plotted in Figure 2.3, resulting from the estimate of the S parameter as defined in Section 2.1 ($\hat{S}$ = 6.3, 95 % CI 5.3 – 7.5). This plot indicates that predictions for CD4 recovery on cART will depend on the time elapsed from seroconversion up until around 3 months, but that the response conditional on baseline CD4 and VL will be stable beyond this point. The fitted functions linking baseline CD4 and the long-term maximum and speed of recovery for both 'early treatment' and 'late treatment' response are plotted in Figure 2.4, and the corresponding influence of the VL random intercept is plotted in Figure 2.5. When treatment is initiated close to the date of seroconversion, the predicted long-term maximum CD4 count for a given baseline value is slightly lower, but the speed of recovery is substantially higher. This is further illustrated through the plotting of predicted median recovery for hypothetical patients in Figure 2.6. The fitted models predict lower long-term CD4 counts beyond 3–5 years, for a given CD4 baseline value, for patients in whom cART is initiated immediately at the time of seroconversion; however, this may be an artefact of the limitation in

the modelling of recovery characteristics in terms of only linear functions of baseline CD4 and VL for this part of the analysis. It should be noted that as CD4 counts decline over time in untreated patients, these results still imply that substantially better CD4 recovery would be expected for patients that initiate treatment close to their date of seroconversion.

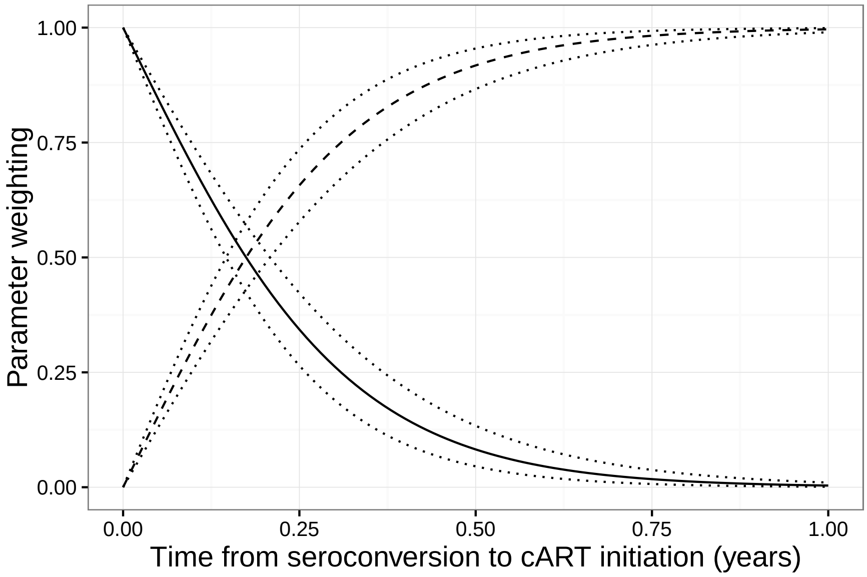

Figure 2.3. Plot of the transition from an 'early treatment' to a 'late treatment' response as estimated for Mod_7'. The weights for the 'early' (——) and 'late' (- - -) parameters linking baseline CD4 and viral load random intercept to CD4 recovery are plotted as a function of the time elapsed from 'true' date of seroconversion to initiation of treatment. 95 % confidence intervals are also plotted (······).

The patient-specific VL random intercept was positively associated with speed of recovery regardless of the time elapsed from seroconversion to treatment initiation, although its relationship with the predicted long-term maximum did differ according to the time to treatment (Figure 2.5). This is further explored through the plotting of predicted median recovery for hypothetical patients in Figure 2.7. Higher viral loads, conditional on the baseline CD4 at treatment initiation, consistently predicted a better recovery in CD4 counts following treatment initiation. It should be noted that the VL random intercept term was found to be negatively correlated both with the CD4 count at seroconversion random intercept ($\hat{r} = -0.28$) and with the slope of CD4 change with respect to time from seroconversion ($\hat{r} = -0.50$), indicating that a high VL is associated with a worse prognosis without treatment. For other patient and drug combination characteristics, the predictions from the model (Figure 2.8) were very similar to those from the model in which the estimated date of seroconversion in each patient was treated as fixed (as shown in Figure 1.5).

The parameters relating to pre-treatment VL measurements fitted in Mod_7' were consistent with previous research on this topic (e.g. Pantazis et al.[5]), with a high av-

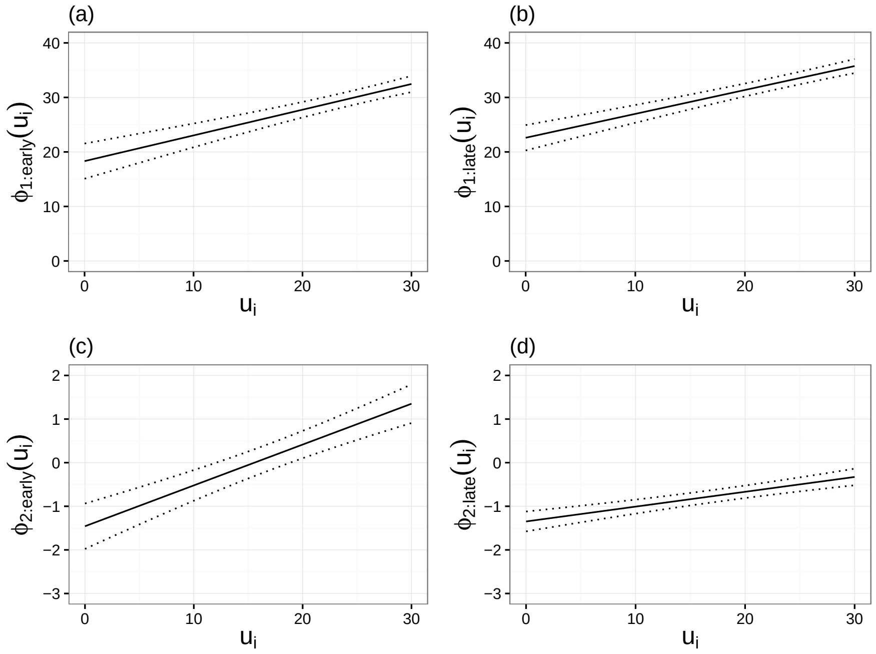

Figure 2.4. Plots of functions linking 'true' baseline CD4 (u_i^+) to post-treatment recovery, $\phi_1(u_i^+)$ (a–b, relating to long-term maximum) and $\phi_2(u_i^+)$ (c–d, relating to speed of response), for Mod_7'. Graphs on the left of each row (a,c) show the fitted functions for 'early treatment' and those on the right (b,d) show the functions 'late treatment'. Pointwise 95 % confidence intervals for the functions are shown (⋯⋯).

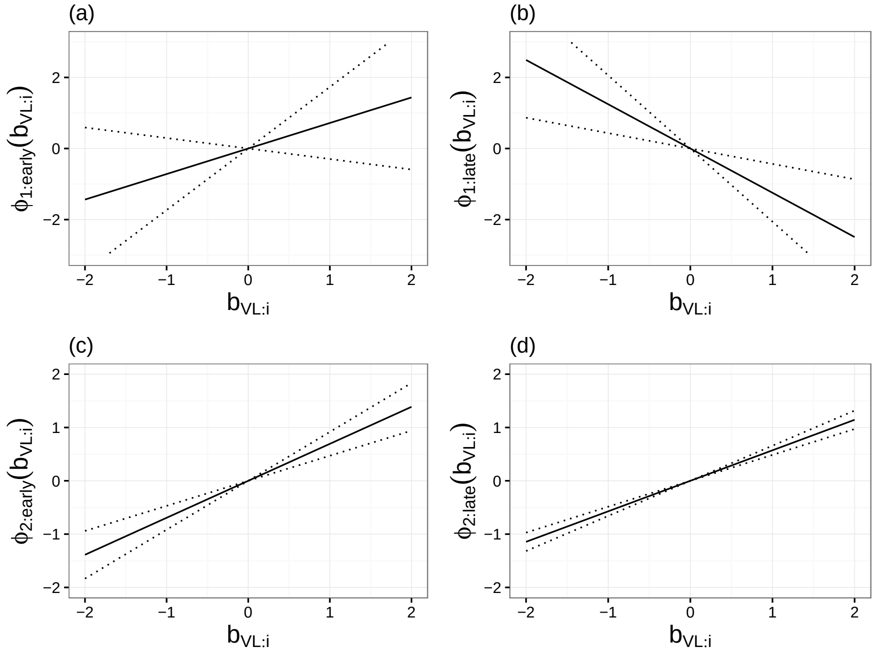

Figure 2.5. Plots of estimated effect of patient-specific viral load random intercept $b_{VL:i}$ on predicted characteristics of post-treatment recovery, $\phi_1(b_{VL:i})$ (a–b, relating to long-term maximum) and $\phi_2(b_{VL:i})$ (c–d, relating to speed of response), for Mod_7'. Graphs on the left of each row (a,c) show the fitted functions for 'early treatment' and those on the right (b,d) show the functions 'late treatment'. Pointwise 95 % confidence intervals for the functions are shown (⋯⋯).

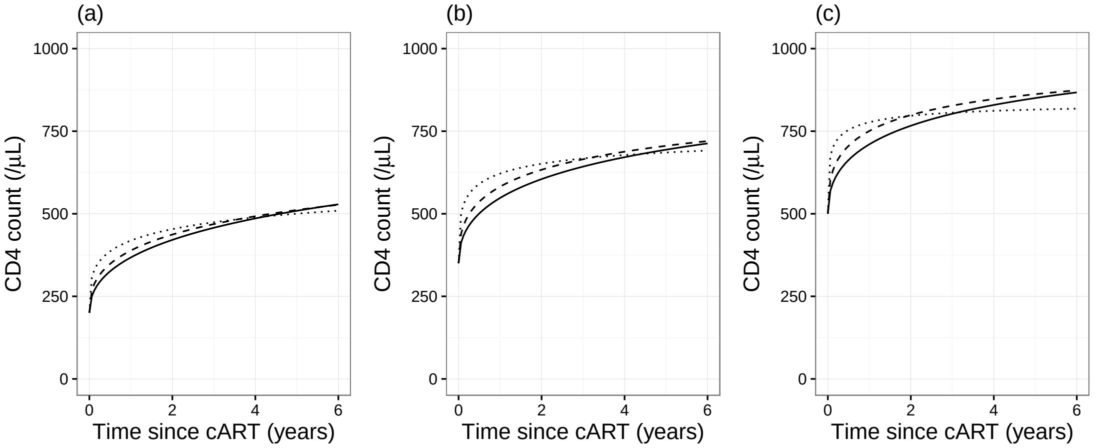

Figure 2.6. Plots of predicted median recovery in CD4 counts, based on Mod_7', for patients with a 'true' baseline value of 200 (a), 350 (b) or 500 (c) cells/μL. Predictions are shown for patients initiating treatment immediately at time of seroconversion (······), at 3 months (- - -) and at 1 year (——). For this plot, all patients are assumed to be men who have sex with men, aged 36 years, with negative test for hepatitis C virus, no prior AIDS diagnosis and starting on a non-nucleoside reverse-transcriptase inhibitor (NNRTI) regimen. They are also assumed to have the population median viral load conditional on time from seroconversion.

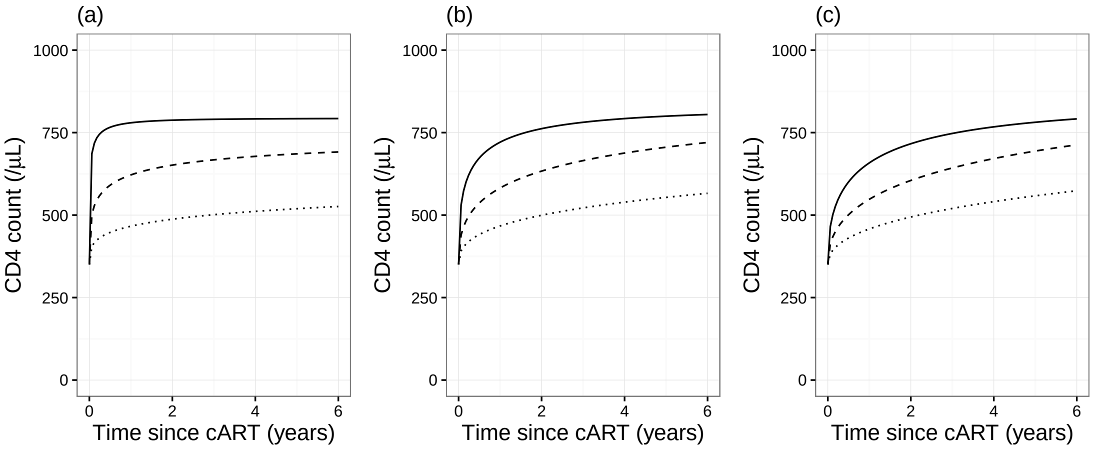

Figure 2.7. Plots of predicted median recovery in CD4 counts, based on Mod_7', for patients with a 'true' baseline value of 350 cells/μL and a patient-specific viral load random intercept (on the $\log_{10}$ scale) corresponding to the 2.5th centile (······, -1.44), 50th centile (- - - , 0) or the 97.5th centile (——, 1.44). Plots are shown of predictions for patients initiating treatment immediately at time of seroconversion (a), at 3 months (b) and at 1 year (c). For this plot, all patients are assumed to be men who have sex with men, aged 36 years, with negative test for hepatitis C virus, no prior AIDS diagnosis and starting on a non-nucleoside reverse-transcriptase inhibitor (NNRTI) regimen.

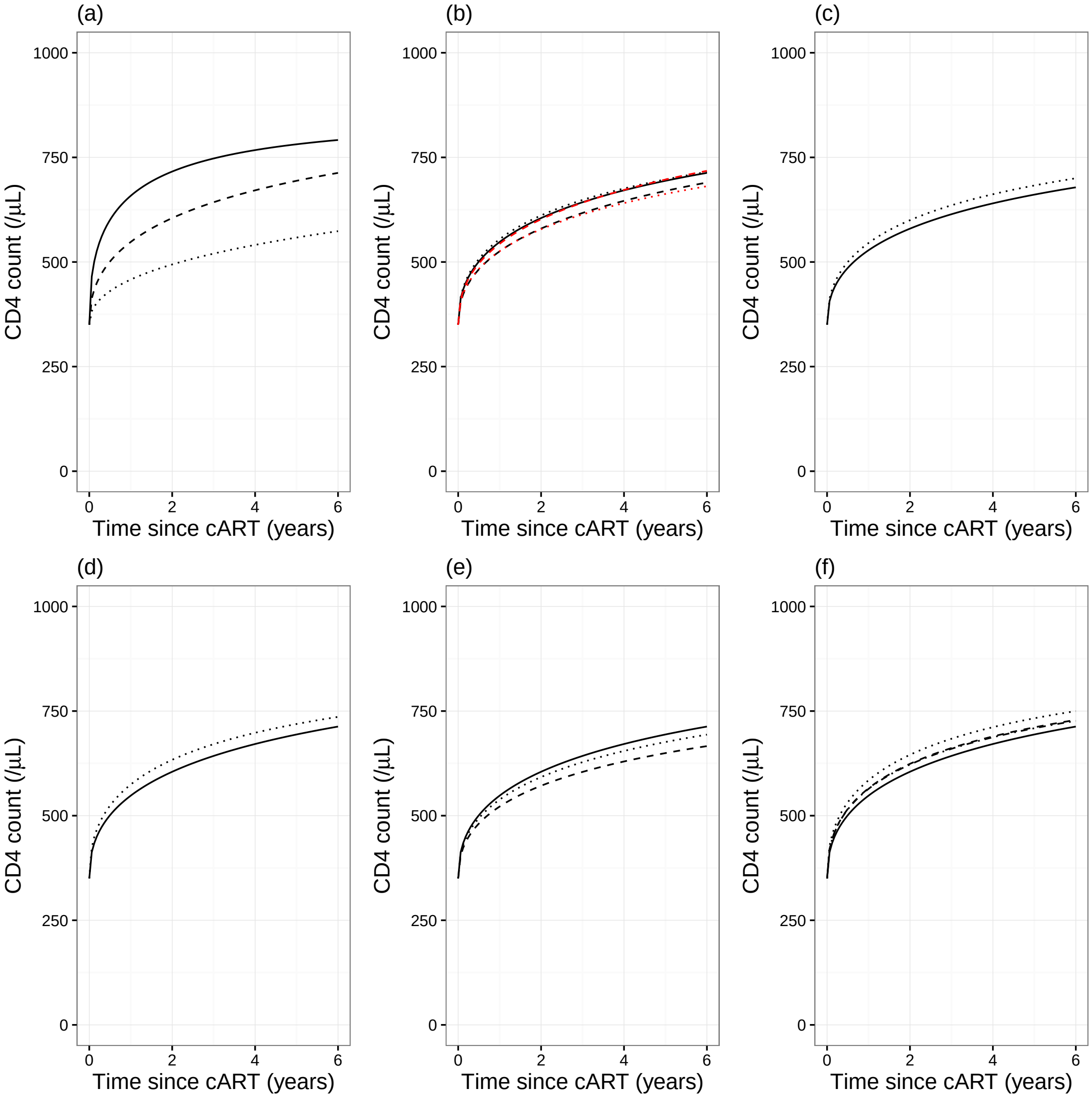

Figure 2.8. Plots of predicted median recovery in CD4 counts, based on Mod'_7, for patients with a 'true' baseline value of 350 according to: (a) patient specific viral load (VL) random intercept (······, 2.5th centile; - - - , 50th centile; ——, 97.5th centile); (b) sex and infection groups (——, men who have sex with men; - - - , male heterosexual; ······, male injecting drug user; - - - , female heterosexual; ······, female injecting drug user); (c) patient age at treatment initiation (······, $age = 20$ years; ——, $age = 60$ years); (d) AIDS diagnosis prior to treatment (······, yes; ——, no); (e) hepatitis C virus (HCV) status (······, no test; - - - , +ve test; ——, -ve test); and (f) cART regimen (······, integrase strand transfer inhibitor; -·-·-, ritonavir-boosted protease inhibitor; - - - , other; ——, non-nucleoside reverse-transcriptase inhibitor (NNRTI)). All patients are assumed to be men who have sex with men, aged 36 years, with negative test for HCV, no prior AIDS diagnosis, median VL and starting on a NNRTI regimen at 1 year since estimated date of seroconversion unless stated otherwise.

erage VL (on the $\log_{10}$ scale) close to the date of seroconversion that drops down to a more stable level after 2–3 months and a gradual increase over time thereafter (Figure 2.9). The estimate of variance for the patient-specific random intercept term ($\widehat{\phi_{VL}} = 0.563$) was larger than the estimate of variance for the examination-specific measurement error ($\widehat{\sigma^2_{VL}} = 0.331$), indicating the presence of consistent differences between patients that were used to link their pre-treatment VL level to the characteristics of CD4 recovery following treatment initiation.

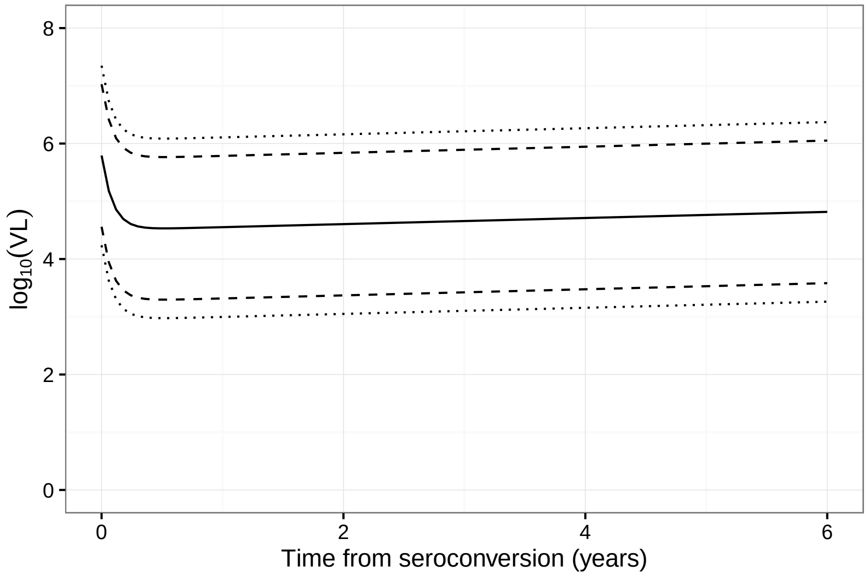

Figure 2.9. Plot of fitted mean viral load (VL, on the $\log_{10}$ scale; ——), as a function of time elapsed since seroconversion in the absence of treatment, resulting from Mod'_7. 5th and 95th percentiles for individual measurements (······) are plotted, and the equivalent percentiles are also shown for the 90 % range of 'true' VL (- - - ; i.e. including between-patient difference relating to the random intercept term in the model, but not examination-specific measurement error).

It was attempted to fit Mod'_7 to a dataset in which CD4 counts were censored if a detectable viral load was observed beyond 6 months after the start of treatment, but model convergence was not achieved. However, Mod'_6 was fitted to this dataset. As shown in Figure 2.10, the predictions made were very similar, although a pre-treatment AIDS diagnosis was no longer associated with any relative improvement in post-treatment recovery. The parameter estimates for Mod'_7 as fitted to the full dataset and for Mod'_6 fitted to the processed dataset with censoring at detectable VL observations are presented in Table 2.2.

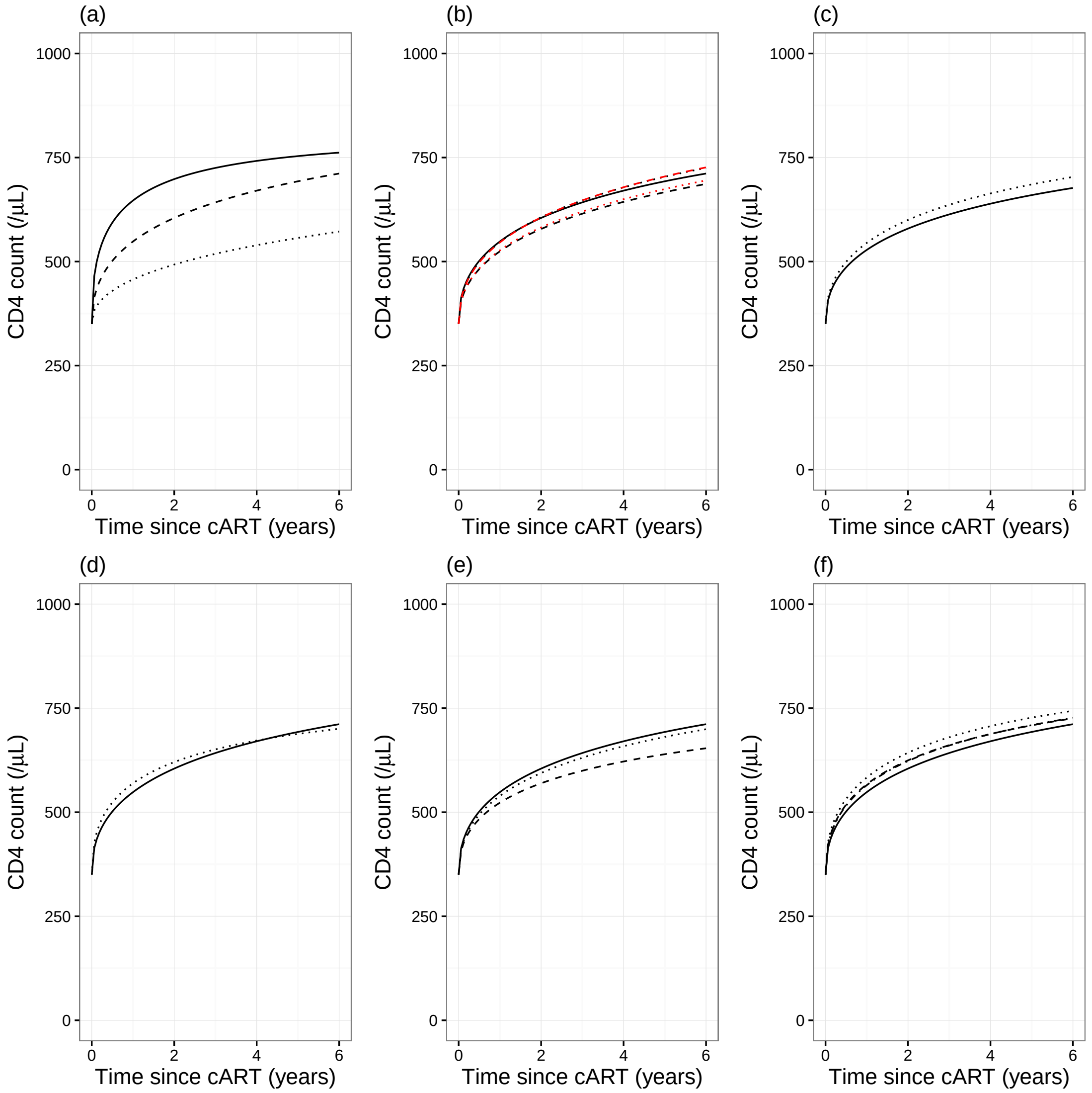

Figure 2.10. Plots of predicted median recovery in CD4 counts, based on Mod_6' fitted to the dataset with censoring at detectable viral load (VL) after 6 months of treatment, for patients with a 'true' baseline value of 350 according to: (a) patient specific viral load (VL) random intercept (······, 2.5^{th} centile; - - - , 50th centile; ——, 97.5^{th} centile); (b) sex and infection groups (——, men who have sex with men; - - - , male heterosexual; ······, male injecting drug user; - - - , female heterosexual; ······, female injecting drug user); (c) patient age at treatment initiation (······, $age = 20$ years; ——, $age = 60$ years); (d) AIDS diagnosis prior to treatment (······, yes; ——, no); (e) hepatitis C virus (HCV) status (······, no test; - - - , +ve test; ——, -ve test); and (f) cART regimen (······, integrase strand transfer inhibitor; -·-·-, ritonavir-boosted protease inhibitor; - - - , other; ——, non-nucleoside reverse-transcriptase inhibitor (NNRTI)). All patients are assumed to be men who have sex with men, aged 36 years, with negative test for HCV, no prior AIDS diagnosis, median VL and starting on a NNRTI regimen at 1 year since estimated date of seroconversion unless stated otherwise.

Table 2.2. Parameter estimates for Mod_7' as applied to the full CASCADE dataset, and for Mod_6' applied to a processed dataset with censoring of CD4 counts at occurrence of detectable viral load (VL) beyond 6 months after treatment initiation.

Para.	Full data fit	Cens data fit	Para. (cont. 1)	Full data fit (cont. 1)	Cens data fit (cont. 1)	Para. (cont. 2)	Full data fit (cont. 2)	Cens data fit (cont. 2)
β_0	22.17 (22.049 to 22.291)	22.184 (22.063 to 22.305)	VA_1	0.718 (-0.295 to 1.732)	0.344 (-0.613 to 1.3)	$AIDS_B$	0.254 (0.062 to 0.446)	0.466 (0.269 to 0.663)
β_1	-1.602 (-1.666 to -1.538)	-1.612 (-1.676 to -1.547)	VA_2	-1.246 (-2.058 to -0.433)	-1.693 (-2.515 to -0.872)	HCV_pre_A	-1.07 (-2.87 to 0.731)	-2.269 (-3.808 to -0.729)
U_{00}	21.743 (20.832 to 22.694)	21.541 (20.632 to 22.489)	VB_1	0.694 (0.47 to 0.917)	1.002 (0.715 to 1.29)	HCV_pre_B	-0.045 (-0.295 to 0.205)	0.113 (-0.112 to 0.338)
ρ	-0.52	-0.52	VB_2	0.572 (0.486 to 0.659)	0.617 (0.532 to 0.702)	$no\_HCV\_test_A$	-0.611 (-1.351 to 0.128)	0.072 (-0.631 to 0.775)
U_{11}	0.658 (0.537 to 0.805)	0.648 (0.527 to 0.797)	MI_A	-0.619 (-3.469 to 2.23)	1.789 (-1.867 to 5.445)	$no\_HCV\_test_B$	0.01 (-0.098 to 0.118)	-0.062 (-0.159 to 0.036)
κ_{pre}	5.688 (5.383 to 6.011)	5.709 (5.398 to 6.039)	MI_B	0.108 (-0.282 to 0.497)	-0.185 (-0.612 to 0.243)	II_A	-0.74 (-1.895 to 0.415)	-0.911 (-2.044 to 0.222)
H_{pre}	0.312 (0.283 to 0.341)	0.315 (0.285 to 0.345)	MH_A	1.278 (0.047 to 2.508)	1.23 (0.087 to 2.373)	II_B	0.29 (0.12 to 0.461)	0.306 (0.138 to 0.473)
σ	1.784 (1.751 to 1.818)	1.781 (1.746 to 1.818)	MH_B	-0.262 (-0.409 to -0.114)	-0.266 (-0.408 to -0.125)	PrI_A	-0.665 (-1.407 to 0.077)	-0.578 (-1.33 to 0.175)
$At1_1$	18.315 (15.09 to 21.54)	15.863 (13.053 to 18.673)	FI_A	0.004 (-3.808 to 3.816)	1.997 (-2.84 to 6.834)	PrI_B	0.168 (0.076 to 0.261)	0.162 (0.069 to 0.254)
$At1_2$	0.472 (0.351 to 0.592)	0.535 (0.427 to 0.642)	FI_B	-0.137 (-0.647 to 0.373)	-0.321 (-0.882 to 0.241)	$other_A$	-0.618 (-2.151 to 0.915)	-0.865 (-2.252 to 0.522)
$At2_1$	22.598 (20.271 to 24.926)	21.258 (18.86 to 23.655)	FH_A	1.262 (0.091 to 2.433)	2.111 (0.955 to 3.266)	$other_B$	0.17 (-0.04 to 0.38)	0.209 (0.019 to 0.399)
$At2_2$	0.438 (0.352 to 0.525)	0.505 (0.41 to 0.601)	FH_B	-0.152 (-0.286 to -0.017)	-0.218 (-0.342 to -0.093)	S	6.298 (5.273 to 7.522)	6.463 (5.26 to 7.94)
$Bt1_1$	-1.458 (-1.978 to -0.937)	-1.28 (-1.989 to -0.571)	$ageA_1$	0.096 (-0.06 to 0.251)	0.092 (-0.064 to 0.247)	β_{0VL}	4.499 (4.477 to 4.521)	4.499 (4.477 to 4.521)
$Bt1_2$	0.094 (0.069 to 0.118)	0.107 (0.075 to 0.138)	$ageA_2$	-0.018 (-0.073 to 0.037)	-0.032 (-0.087 to 0.023)	β_{1VL}	0.053 (0.047 to 0.059)	0.053 (0.047 to 0.059)
$Bt2_1$	-1.348 (-1.576 to -1.12)	-1.232 (-1.48 to -0.984)	$ageA_3$	0.047 (-0.107 to 0.201)	0.084 (-0.068 to 0.236)	β_{2VL}	1.295 (1.23 to 1.359)	1.29 (1.225 to 1.355)
$Bt2_2$	0.034 (0.024 to 0.044)	0.028 (0.016 to 0.041)	$ageA_4$	-0.047 (-0.196 to 0.102)	-0.075 (-0.22 to 0.071)	β_{3VL}	10.832 (9.704 to 12.091)	10.819 (9.686 to 12.085)
P	9.788 (8.248 to 11.615)	10.441 (8.974 to 12.148)	$ageB_1$	-0.004 (-0.027 to 0.019)	-0.001 (-0.023 to 0.022)	ϕ_{VL}	0.563 (0.541 to 0.586)	0.561 (0.539 to 0.584)
κ_{post}	4.894 (4.618 to 5.187)	4.201 (3.907 to 4.516)	$ageB_2$	-0.001 (-0.008 to 0.007)	0 (-0.008 to 0.007)	σ_{VL}	0.575 (0.57 to 0.58)	0.575 (0.57 to 0.579)
H_{post}	0.236 (0.22 to 0.253)	0.214 (0.194 to 0.233)	$ageB_3$	0.002 (-0.019 to 0.024)	0.002 (-0.019 to 0.022)	$\rho_{VL:CD4_0}$	-0.28	-0.28
D_{t1}/D	0.301 (0.26 to 0.348)	0.438 (0.421 to 0.455)	$ageB_4$	-0.002 (-0.022 to 0.018)	-0.002 (-0.022 to 0.017)	$\rho_{VL:CD4_1}$	-0.50	-0.51
D_{t2}	0.441 (0.424 to 0.459)	—	$AIDS_A$	-0.889 (-2.374 to 0.596)	-2.581 (-3.977 to -1.184)			

95% CIs are given in parentheses. Parameters (Para.) are defined as in Table 1.2 unless stated otherwise. $At1_1$ and $At1_2$ are the intercept and slope of the function linking baseline CD4 to long-term maximum for 'early treatment', while $At2_1$ and $At2_2$ are the equivalent parameters for 'late treatment'. $Bt1_1$ and $Bt1_2$, and $Bt2_1$ and $Bt2_2$ are the corresponding parameters for rate of recovery. D_{t1} and D_{t2} are the shape parameters for the Janoshek–Sager curve corresponding to 'early treatment' and 'late treatment', respectively, with D the single parameter for Mod_6'. VA_{t1} and VB_{t1} represent the effect of the patient specific viral load (VL) random intercept on the long-term maximum and speed of recovery for 'early treatment', while VA_{t2} and VB_{t2} are the equivalent parameters for 'late treatment'. β_{0VL}, β_{1VL}, β_{2VL} and β_{3VL} relate to the population median VL as described in Section 2.2, and ϕ_{VL} and σ_{VL} denote the random intercept variance and VL measurement error SD. $\rho_{VL:CD4_0}$ and $\rho_{VL:CD4_1}$ are the correlations between VL random intercept and CD4 random intercept and slope, respectively.

3 Residual plots and further sensitivity analyses

To check the fundamental modelling assumptions, we present here an analysis of diagnostic residual plots for Mod_4 as described in Section 1 (without censoring at virological failure). Cholesky-transformed residuals for CD4 counts in the pre- and post-treatment periods were calculated as described in Section 2 of the supplementary Appendix for Stirrup *et al.*[1], ignoring for now the patient-specific latent scaling variables for the fractional Brownian motion processes. The transformed residuals plotted against time show approximately symmetric distributions centred on zero as expected for both the pre- (Figure 3.1) and post-treatment (Figure 3.2) periods. However, quantile–quantile plots relative to the expected standard normal distribution display heavy tails (Figures 3.3 and 3.4).

In order to explore the potential importance of the observed deviation from modelling assumptions (i.e. the presence of a heavy-tailed distribution for the transformed residuals), we refitted Mod_4 allowing between-patient differences in the variability of CD4 counts over time. This was implemented by adding correlated gamma-distributed patient-specific scaling variables for the pre- and post-treatment fractional Brownian motion processes as described by Stirrup *et al.*[1]. The model was fitted by maximum likelihood estimation using the Laplace approximation for the marginal log-likelihood. Cholesky-transformed residuals were then evaluated for this extended model based on the posterior predictive modes of the scaling variables for each patient ($\hat{w}_{1:i}$ and $\hat{w}_{2:i}$). Once again plots of residuals against time displayed a symmetric distribution around zero (Figures 3.5 and 3.6), and quantile–quantile plots now showed minimal deviation from the expected straight line (Figures 3.7 and 3.8).

Convergence of parameter estimates was not achieved when an equivalent extended model was fitted for Mod_{10}. Mod_4 includes linear effects for baseline CD4 count stratified by time to treatment initiation and additional effects of baseline VL, using a natural cubic spline basis, on the characteristics of response to treatment. The relationships between these predictive factors and response to treatment indicated by the resulting extended Mod_4 were nearly identical to those described for Mod_{10}; this is illustrated by Figures 3.9 and 3.10. The spread of the 5th and 95th centiles of predictions was also similar to the models that did not take between-patient differences in variability into account.

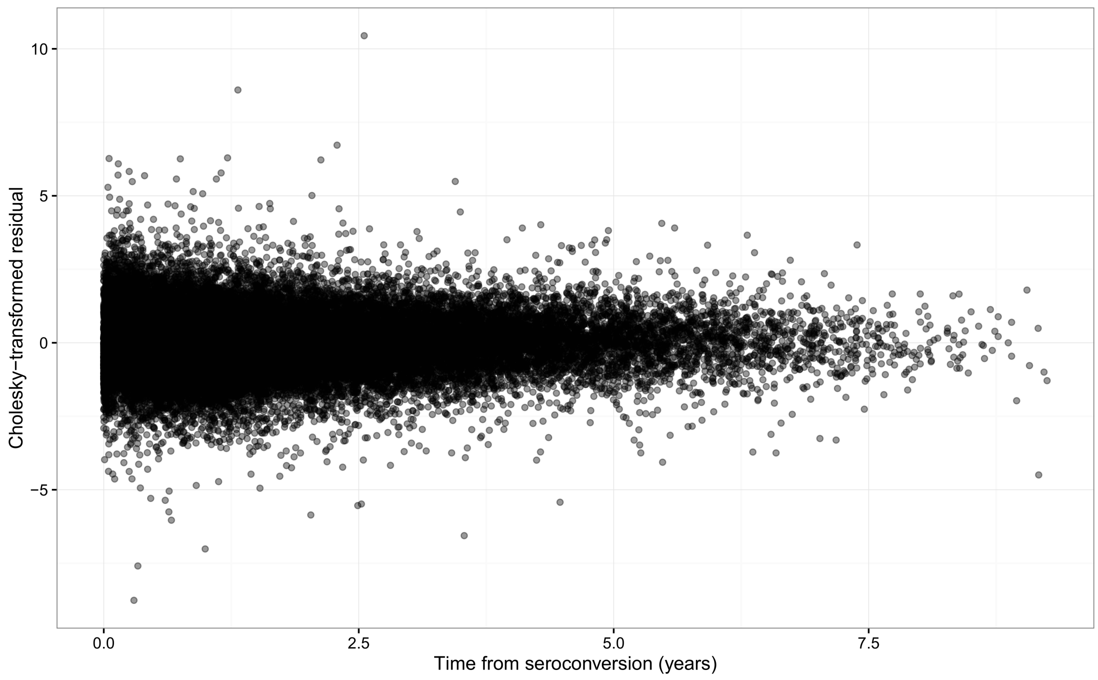

Figure 3.1. Plot of Cholesky-transformed residuals of pre-treatment observations for Mod_4 against time from seroconversion.

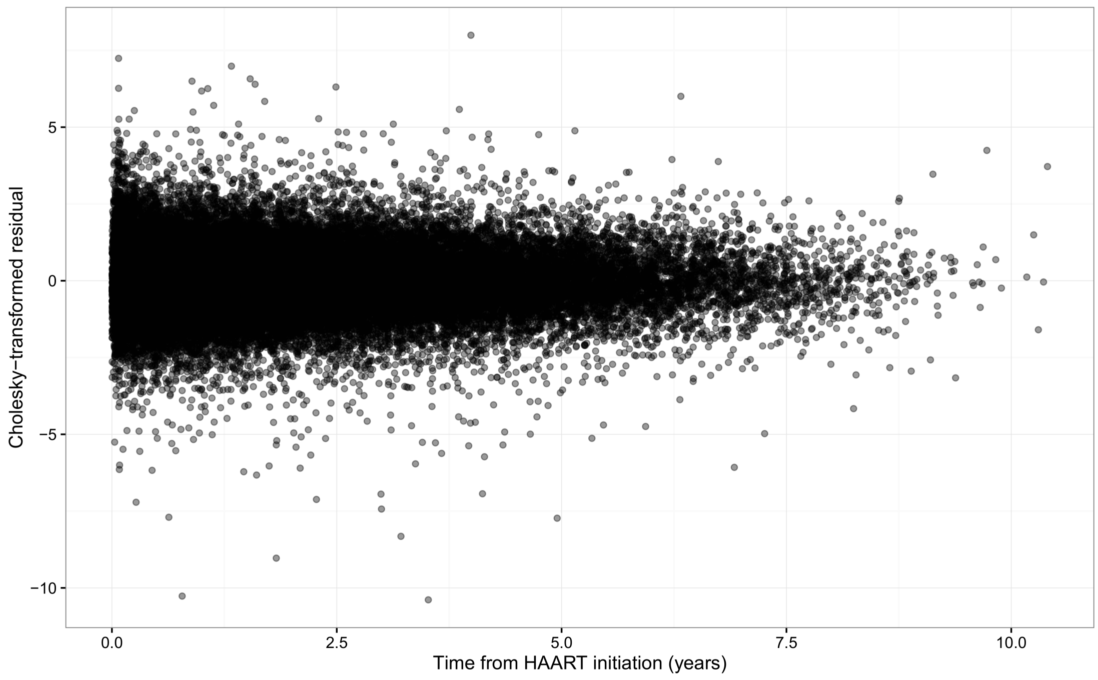

Figure 3.2. Plot of Cholesky-transformed residuals of post-treatment observations for Mod_4 against time from initiation of HAART, calculated using the posterior predictive mode of the baseline CD4 value $\hat{u}_i$.

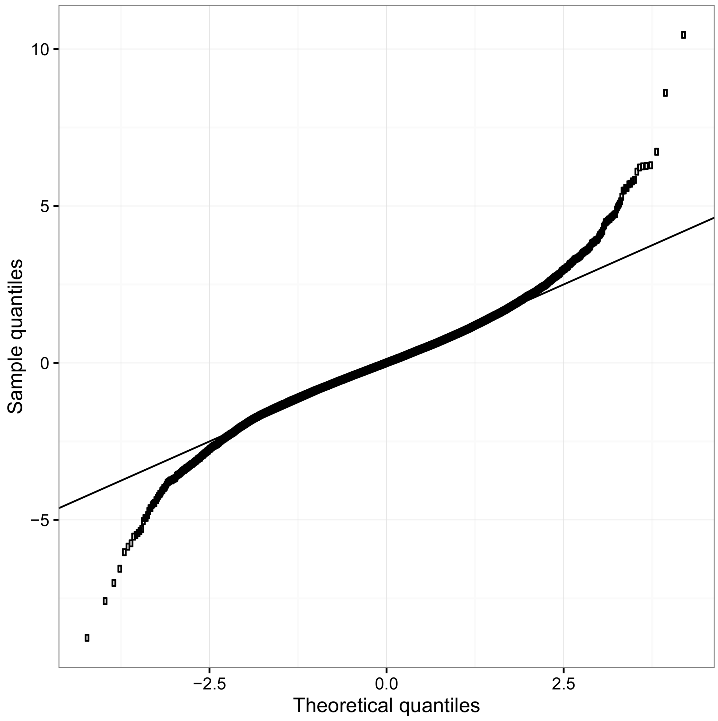

Figure 3.3. Quantile–quantile plot of Cholesky-transformed residuals of pre-treatment observations for Mod_4.

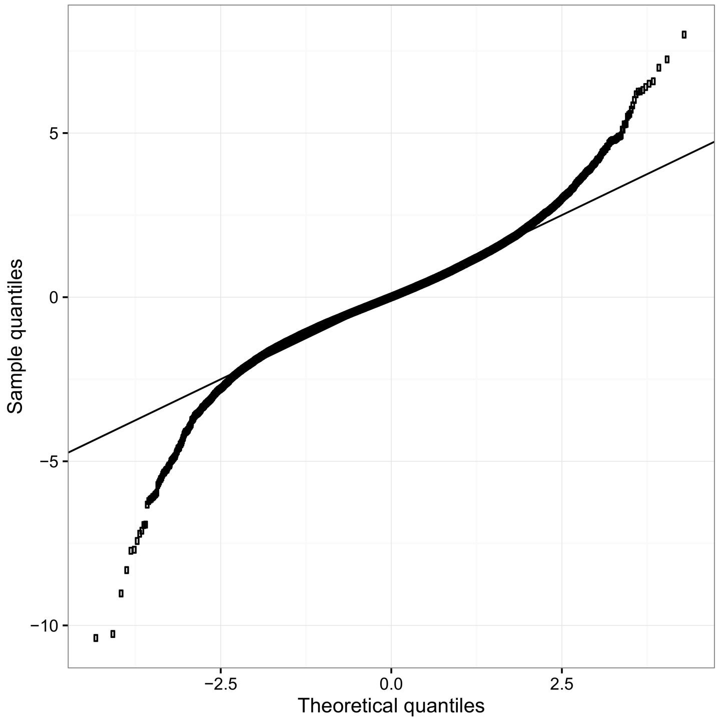

Figure 3.4. Quantile–quantile plot of Cholesky-transformed residuals of post-treatment observations for Mod_4, calculated using the posterior predictive mode of the baseline CD4 value $\hat{u}_i$.

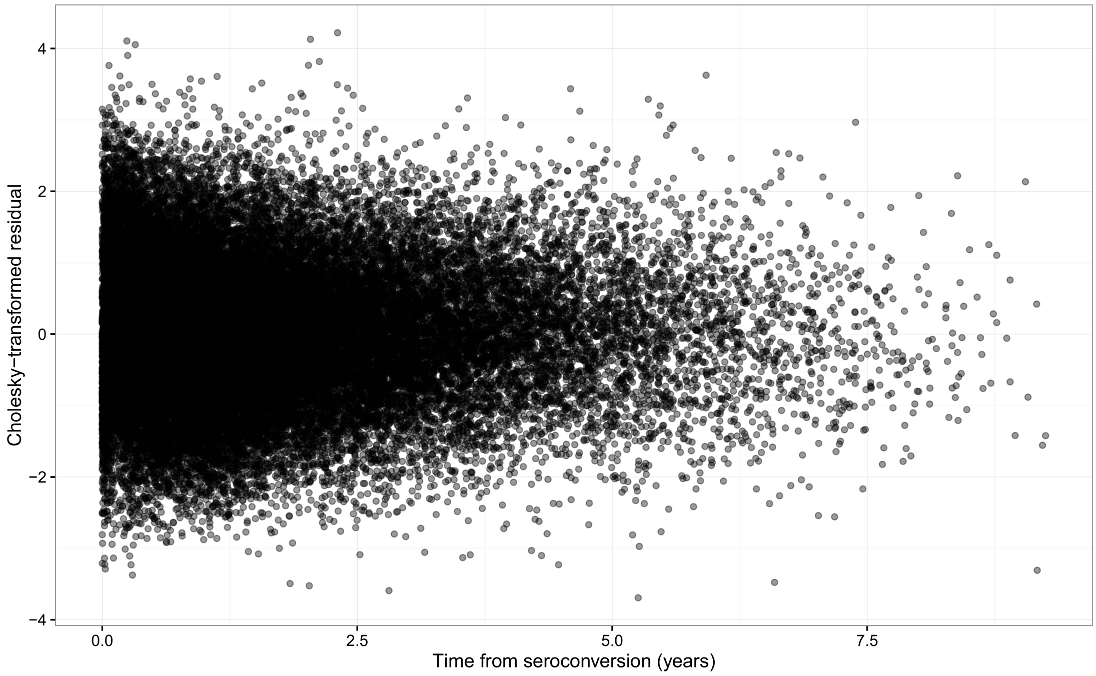

Figure 3.5. Plot of Cholesky-transformed residuals of pre-treatment observations for the extended version of Mod_4 against time from seroconversion, calculated using the posterior predictive mode of the latent scaling variable $\hat{w}_{1:i}$ for each patient.

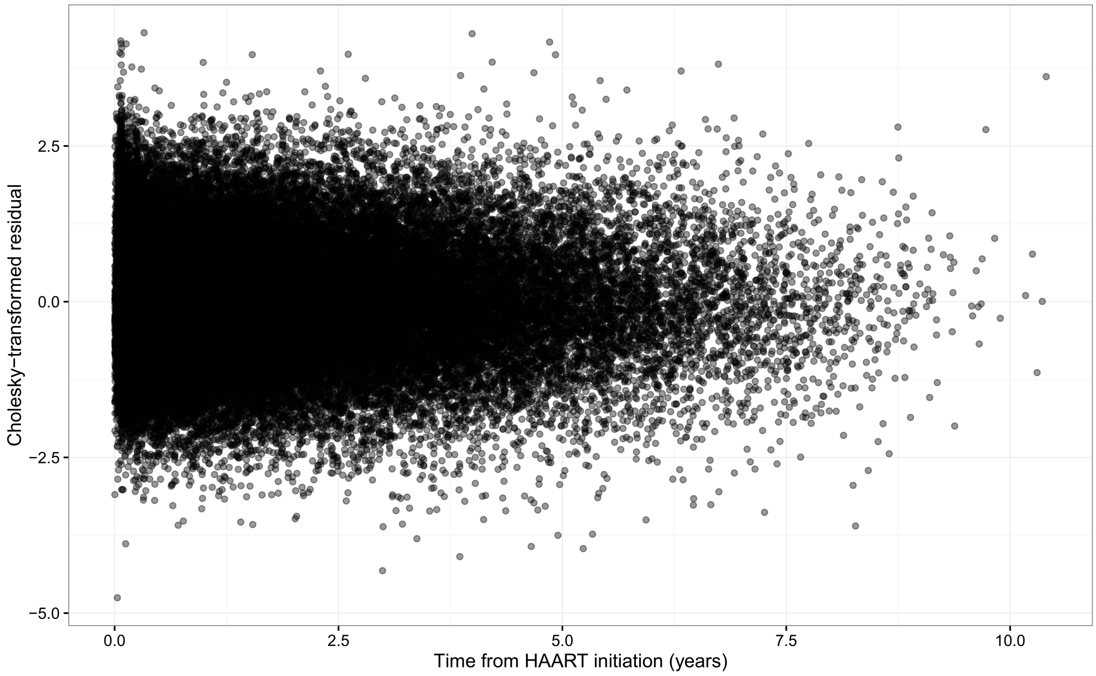

Figure 3.6. Plot of Cholesky-transformed residuals of post-treatment observations for the extended version of Mod_4 against time from initiation of HAART, calculated using the posterior predictive mode of the baseline CD4 value $\hat{u}_i$ and the latent scaling variable $\hat{w}_{2:i}$ for each patient.

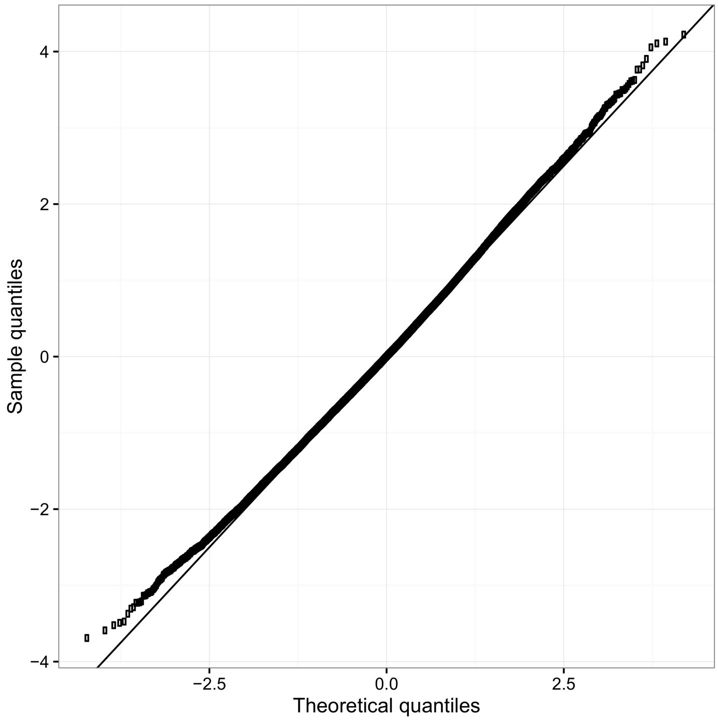

Figure 3.7. Quantile–quantile plot of Cholesky-transformed residuals of pre-treatment observations for the extended version of Mod_4, calculated using the posterior predictive mode of the latent scaling variable $\hat{w}_{1:i}$ for each patient.

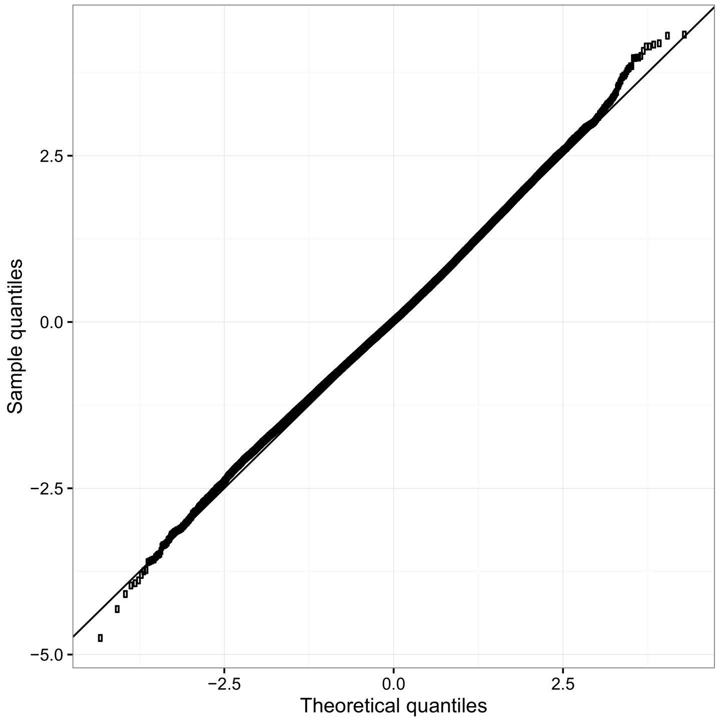

Figure 3.8. Quantile–quantile plot of Cholesky-transformed residuals of post-treatment observations for the extended version of Mod_4, calculated using the posterior predictive mode of the baseline CD4 value $\hat{u}_i$ and the latent scaling variable $\hat{w}_{2:i}$ for each patient.

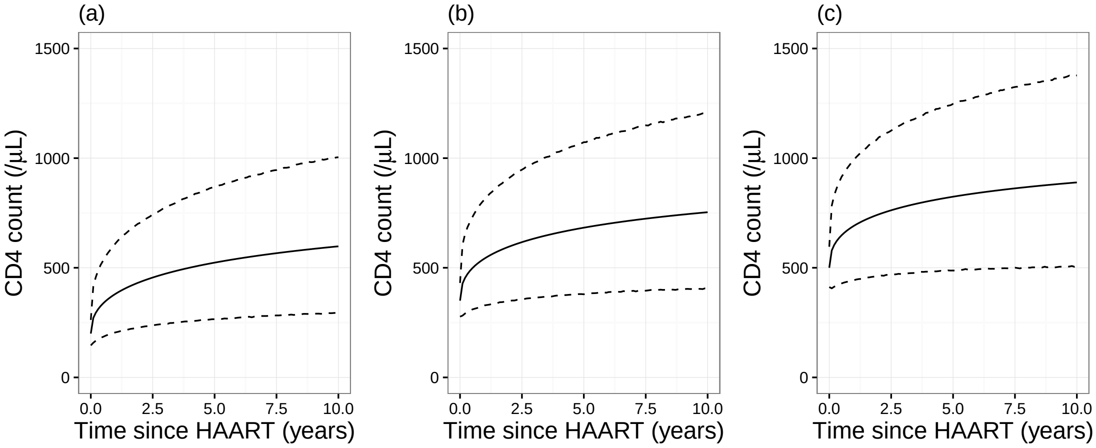

Figure 3.9. Plots of predicted median (——) and 5th and 95th centiles (- - -) for recovery in CD4 counts, based on Mod_4 with the model extended using linked multivariate-t distributions for the pre- and post-treatment stochastic process components, for patients with a 'true' baseline value of 200 (a), 350 (b) or 500 (c) cells/μL. For this plot, viral load prior to treatment is fixed at the overall $\log_{10}$ median of 4.825 and treatment initiation is set to be beyond 1 year from seroconversion. The marginal distribution is assumed for the latent scaling variable for the fractional Brownian motion process, i.e. without conditioning on any potential pre-treatment information, and the combination of multivariate normal and t distributions is approximated through averaging over 1000 draws from the relevant gamma distribution. The pattern of predictions is very close to those resulting from Mod_{10} as displayed in Figure 1.6.

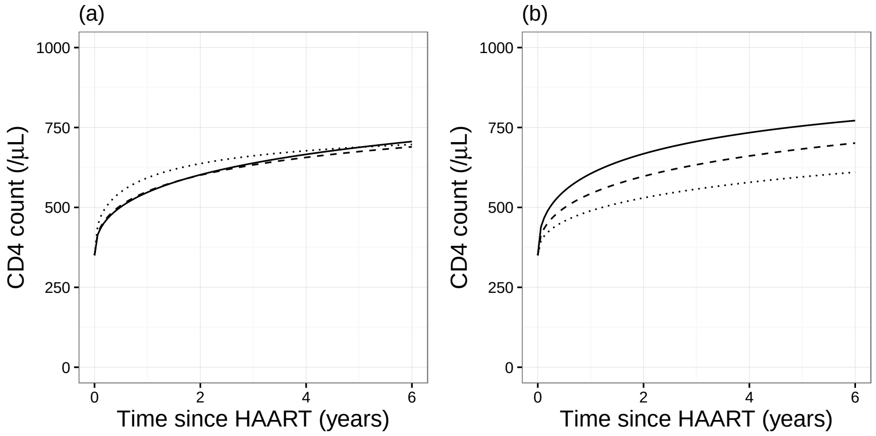

Figure 3.10. Plots of predicted median recovery in CD4 counts, based on Mod_4 with the model extended using linked multivariate-t distributions for the pre- and post-treatment stochastic process components, for patients with a 'true' baseline value of 350 according to: (a) treatment initiation within 6 months of seroconversion (······), beyond 6 months but within 1 year (- - -) and for patients who started treatment beyond 1 year (——) and (b) with viral load (VL) prior to treatment initiation (······, $\log_{10}$(VL) = 2.7; - - - , $\log_{10}$(VL) = 4.7; ——, $\log_{10}$(VL) = 5.7). For (a) VL prior to treatment is fixed at the overall $\log_{10}$ median of 4.825 and for (b) treatment initiation is set to be greater than 1 year from seroconversion. The pattern of predictions is very similar to those resulting from Mod_{10} as displayed in Figures 1.4b and 1.5a.

4 Further Discussion regarding 'speed of response' to treatment

The strong positive association of baseline CD4 count with the long-term maximum of post-treatment recovery found in our analysis was expected given the findings of previous research on this topic[15–19]. However, there has been less of a consensus in the literature regarding the relationship between baseline CD4 count and the initial speed of recovery. Smith *et al.*[20] reported that higher baseline values were associated with both lower observed post-treatment increases at 3 months and a lower rate of increase beyond this point in time, and Hunt *et al.*[21] also reported greater gains in patients with lower baseline values, particularly within the first 2 years after initiation of treatment. However, Florence *et al.*[22] reported that lower baseline CD4 counts were predictive of a poor response within 6–12 months of cART initiation, and Moore *et al.*[23] also found greater increases in CD4 cell count at 6 months post-treatment for higher baseline values up to 350 cells/μL.

In the framework that we have developed, the model term relating to 'speed of recovery' following treatment initiation represents the speed of transition from the baseline state to the long-term maximum for any given patient, rather than the rate of increase in terms of the CD4 count itself. As such, the models that we have fitted indicate that the absolute rate of increase in CD4 count will be lower for patients with 'true' baseline CD4 counts above around 600 cells/μL, due to the fact that there is less of a difference between the baseline value and the long-term maximum in such cases. However, this cannot wholly explain the inconsistency in papers in the literature regarding this topic as these have largely described patients with lower CD4 counts at initiation of cART, for example Smith *et al.*[20] report a median (IQR) pre-treatment CD4 count of 194 (75–314) cells/μL, with very similar values of 195 (118–274) cells/μL reported by Florence *et al.*[22]. For 'true' baseline CD4 counts below 600 cells/μL, our models predict a positive relationship between the baseline value and the rate of post-treatment increase in absolute terms. As such, we suggest that the inconsistencies observed could be due to the fact that lower observed baseline CD4 counts are likely to be more strongly downwardly biased as a result of selective treatment initiation and 'regression to the mean'-type effects, which will result in an increase in the apparent response to treatment, particularly at the first observation for each patient; the degree of bias is dependent on the observation and treatment initiation schedules applied to any given cohort, and so this could explain the observed differences in findings between studies.

When considering differences in initial increase by baseline CD4 count, one should keep in mind that the character of such interaction depends on the scale at which it is quantified. Geskus *et al.*[24] have previously argued that it is better to assess changes

in CD4 count on the cube-root scale, which they found to be more linearly associated with the risk of AIDS than the original CD4 value.

References

[1] Stirrup OT, Babiker AG, and Copas AJ. Combined models for pre- and post-treatment longitudinal biomarker data: an application to CD4 counts in HIV-patients. *BMC Medical Research Methodology*, 16:121, 2016.

[2] Janoschek A. Das reaktionshinetische grundgesetz und seire beziehungen zum wachstumsund ertragsgesetz. *Statistische Vierteljahresschrift*, 10:25–37, 1957.

[3] Sager G. Seasonally modified forms of the revised Janoschek growth function. *Gegenbaurs morphologisches Jahrbuch, Leipzig*, 130:659–669, 1984.

[4] Panik MJ. *Growth Curve Modeling: Theory and Applications*, chapter Parametric Growth Curve Modeling. Hoboken, NJ: Wiley, 2014.

[5] Pantazis N, Touloumi G, Walker AS, and Babiker AG. Bivariate modelling of longitudinal measurements of two human immunodeficiency type 1 disease progression markers in the presence of informative drop-outs. *Journal of the Royal Statistical Society: Series C (Applied Statistics)*, 54:405–423, 2005.

[6] Muñoz A, Carey V, Taylor JM, Chmiel JS, Kingsley L, Van Raden M, and Hoover DR. Estimation of time since exposure for a prevalent cohort. *Statistics in Medicine*, 11:939–952, 1992.

[7] Geskus RB. On the inclusion of prevalent cases in HIV/AIDS natural history studies through a marker-based estimate of time since seroconversion. *Statistics in Medicine*, 19:1753–1769, 2000.

[8] Taffé P, May M, and Swiss HIV Cohort Study. A joint back calculation model for the imputation of the date of HIV infection in a prevalent cohort. *Statistics in Medicine*, 27:4835–4853, 2008.

[9] Sommen C, Commenges D, Vu SL, Meyer L, and Alioum A. Estimation of the distribution of infection times using longitudinal serological markers of HIV: implications for the estimation of HIV incidence. *Biometrics*, 67:467–475, 2011.

[10] Drylewicz J, Guedj J, Commenges D, and Thiébaut R. Modeling the dynamics of biomarkers during primary HIV infection taking into account the uncertainty of infection date. *The Annals of Applied Statistics*, 4:1847–1870, 2010.

[11] Skaug H and Fournier D. *Random Effects in AD Model Builder: ADMB-RE User Guide*, chapter Random Effects Modeling. ADMB Foundation: Honolulu, version 11.4 edition, 2015.

[12] Thiébaut R and Jacqmin-Gadda H. Mixed models for longitudinal left-censored repeated measures. *Computer Methods and Programs in Biomedicine*, 74:255–260, 2004.

[13] Thiébaut R, Jacqmin-Gadda H, Babiker A, Commenges D, and CASCADE Collaboration. Joint modelling of bivariate longitudinal data with informative dropout and left-censoring, with application to the evolution of CD4+ cell count

and HIV RNA viral load in response to treatment of HIV infection. *Statistics in Medicine*, 24:65–82, 2005.

[14] Tobin J. Estimation of relationships for limited dependent variables. *Econometrica*, 26:24–36, 1958.

[15] Kaufmann GR, Perrin L, Pantaleo G, Opravil M, Furrer H, Telenti A, Hirschel B, Ledergerber B, Vernazza P, Bernasconi E, Rickenbach M, Egger M, Battegay M, and Swiss HIV Cohort Study Group. CD4 T-lymphocyte recovery in individuals with advanced HIV-1 infection receiving potent antiretroviral therapy for 4 years: the Swiss HIV Cohort Study. *Archives of Internal Medicine*, 163:2187–2195, 2003.

[16] Moore RD and Keruly JC. CD4+ cell count 6 years after commencement of highly active antiretroviral therapy in persons with sustained virologic suppression. *Clinical Infectious Diseases*, 44:441–446, 2007.

[17] Lok JJ, Bosch RJ, Benson CA, Collier AC, Robbins GK, Shafer RW, Hughes MD, and ALLRT team. Long-term increase in CD4+ T-cell counts during combination antiretroviral therapy for HIV-1 infection. *AIDS*, 24:1867–1876, 2010.

[18] Hughes RA, Sterne JA, Walsh J, Bansi L, Gilson R, Orkin C, Hill T, Ainsworth J, Anderson J, Gompels M, Dunn D, Johnson MA, Phillips AN, Pillay D, Leen C, Easterbrook P, Gazzard B, Fisher M, and Sabin CA. Long-term trends in CD4 cell counts and impact of viral failure in individuals starting antiretroviral therapy: UK Collaborative HIV Cohort (CHIC) study. *HIV Medicine*, 12:583–593, 2011.

[19] Gras L, Kesselring AM, Griffin JT, van Sighem AI, Fraser C, Ghani AC, Miedema F, Reiss P, Lange JM, and de Wolf F; ATHENA and Netherlands National Observational Cohort Study. CD4 cell counts of 800 cells/mm3 or greater after 7 years of highly active antiretroviral therapy are feasible in most patients starting with 350 cells/mm3 or greater. *Journal of Acquired Immune Deficiency Syndromes*, 45:183–192, 2007.

[20] Smith CJ, Sabin CA, Youle MS, Kinloch de Loes S, Lampe FC, Madge S, Cropley I, Johnson MA, and Phillips AN. Factors influencing increases in CD4 cell counts of HIV-positive persons receiving long-term highly active antiretroviral therapy. *The Journal of Infectious Diseases*, 190:1860–1868, 2004.

[21] Hunt PW, Deeks SG, Rodriguez B, Valdez H, Shade SB, Abrams DI, Kitahata MM, Krone M, Neilands TB, Brand RJ, Lederman MM, and Martin JN. Continued CD4 cell count increases in HIV-infected adults experiencing 4 years of viral suppression on antiretroviral therapy. *AIDS*, 17:1907–1915, 2003.

[22] Florence E, Lundgren J, Dreezen C, Fisher M, Kirk O, Blaxhult A, Panos G, Katlama C, Vella S, and Phillips A; EuroSIDA Study Group. Factors associated with a reduced CD4 lymphocyte count response to HAART despite full viral suppression in the EuroSIDA study. *HIV Medicine*, 4:255–262, 2003.

[23] Moore DM, Harris R, Lima V, Hogg B, May M, Yip B, Justice A, Mocroft A, Reiss P, Lampe F, Chêne G, Costagliola D, Elzi L, Mugavero MJ, Monforte AD, Sabin C, Podzamczer D, Fätkenheuer G, Staszewski S, Gill J, and Sterne JA; Antiretroviral Therapy Cohort Collaboration. Effect of baseline CD4 cell counts on the clinical significance of short-term immunologic response to antiretroviral therapy in individuals with virologic suppression. *Journal of Acquired Immune Deficiency Syndromes*, 52:357–363, 2009.

[24] Geskus RB, Prins M, Hubert J-B, Miedema F, Berkhout B, Rouzioux C, Delfraissy J-F, and Meyer L. The HIV RNA setpoint theory revisited. *Retrovirology*, 4(1):65, 2007.
